# Rnd3 protects against doxorubicin-induced cardiotoxicity through inhibition of PANoptosis in a Rock1/Drp1/mitochondrial fission-dependent manner

**DOI:** 10.1038/s41419-024-07322-0

**Published:** 2025-01-04

**Authors:** Wen Ge, Xiaohua Zhang, Jie Lin, Yangyang Wang, Xiao Zhang, Yu Duan, Xinchun Dai, Jiye Zhang, Yan Zhang, Mengyuan Jiang, Huanhuan Qiang, Zhijing Zhao, Xuebin Zhang, Dongdong Sun

**Affiliations:** Department of Cardiology, Xijing Hospital, Fourth Military Medical University, Xi’an, Shaanxi China

**Keywords:** Cell death, Heart failure

## Abstract

Doxorubicin, a representative drug of the anthracycline class, is widely used in cancer treatment. However, Doxorubicin-induced cardiotoxicity (DIC) presents a significant challenge in its clinical application. Mitochondrial dysfunction plays a central role in DIC, primarily through disrupting mitochondrial dynamics. This study aimed to investigate the impact of Rnd3 (a Rho family GTPase 3) on DIC, with a focus on mitochondrial dynamics. Cardiomyocyte-specific Rnd3 transgenic mice (Rnd3-Tg) and Rnd3^LSP/LSP^ mice (N-Tg) were established for in vivo experiments, and adenoviruses harboring Rnd3 (Ad-Rnd3) or negative control (Ad-Control) were injected in the myocardium for in vitro experiments. The DIC model was established using wild-type, N-Tg, and Rnd3-Tg mice, with subsequent intraperitoneal injection of Dox for 4 weeks. The molecular mechanism was explored through RNA sequencing, immunofluorescence staining, co-immunoprecipitation assay, and protein-protein docking. Dox administration induced significant mitochondrial injury and cardiac dysfunction, which was ameliorated by Rnd3 overexpression. Further, the augmentation of Rnd3 expression mitigated mitochondrial fragmentation which is mediated by dynamin-related protein 1 (Drp1), thereby ameliorating the PANoptosis (pyroptosis, apoptosis, and necroptosis) response induced by Dox. Mechanically, the interaction between Rnd3 and Rho-associated kinase 1 (Rock1) may impede Rock1-induced Drp1 phosphorylation at Ser616, thus inhibiting mitochondrial fission and dysfunction. Interestingly, Rock1 knockdown nullified the effects of Rnd3 on cardiomyocytes PANoptosis, as well as Dox-induced cardiac remodeling and dysfunction elicited by Rnd3. Rnd3 enhances cardiac resilience against DIC by stabilizing mitochondrial dynamics and reducing PANoptosis. Our findings suggest that the Rnd3/Rock1/Drp1 signaling pathway represents a novel target for mitigating DIC, and modulating Rnd3 expression could be a strategic approach to safeguarding cardiac function in patients undergoing Dox treatment.

**Figure:**
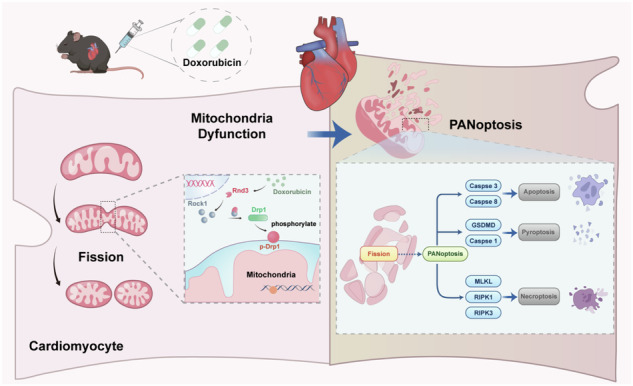
The graphical abstract illustrated the cardioprotective role of Rnd3 in DIC. Rnd3 directly binds to Rock1 in cytoplasm and ameliorates mitochondrial fission by inhibiting Drp1 phosphorylation at ser616, thereby alleviating PANoptosis (apoptosis, pyroptosis, and necroptosis) in DIC.

The graphical abstract illustrated the cardioprotective role of Rnd3 in DIC. Rnd3 directly binds to Rock1 in cytoplasm and ameliorates mitochondrial fission by inhibiting Drp1 phosphorylation at ser616, thereby alleviating PANoptosis (apoptosis, pyroptosis, and necroptosis) in DIC.

## Introduction

Doxorubicin, which is a representative drug of the anthracycline class, is a potent chemotherapeutic agent for its efficacy against a broad spectrum of cancers [1, 2]. However, its clinical application is significantly hampered by the onset and development of cardiotoxicity [3]. Doxorubicin-induced cardiotoxicity (DIC) not only limits its therapeutic dosage but also poses long-term threat to cancer survivors [4, 5]. Thus, there is an urgent need to elucidate the underlying mechanisms of DIC and mitigate their adverse effects.

Mitochondrial dysfunction serves as a cornerstone in the pathology of DIC, orchestrating a cascade of detrimental effects within cardiomyocytes [6, 7]. Such defect arises from disruption of mitochondrial dynamics, a critical equilibrium between mitochondrial fission and fusion [7]. Perturbation of this delicate balance leads to mitochondrial fragmentation, a hallmark of cellular distress, contributing directly to cardiomyocyte death and subsequent cardiac dysfunction [8, 9]. Modulation of mitochondrial dynamics presents a promising strategy for reducing or even reversing cardiotoxic sequelae of Dox [7], thereby safeguarding cardiac function of patients receiving Dox treatment.

Apoptosis, collectively termed ‘PANoptosis’ together with other forms of programmed cell death such as pyroptosis and necroptosis [10], has been identified as a critical modality in DIC [11]. Abnormal mitochondrial fission, regulated by dynamin-related protein 1 (Drp1), often precedes cell death and acts as a pivotal link between mitochondrial dysfunction and activation of cell death pathways [12–14]. Excessive mitochondrial fission triggered by Dox exposure can initiate programmed cell death through inducing mitochondrial fragmentation [15], loss of mitochondrial membrane potential, and release of pro-apoptotic factors. Importantly, the interplay between mitochondrial fission and these forms of programmed cell death may present a critical aspect of the pathogenesis of DIC, implicating the necessity of a comprehensive approach to protect against DIC.

Rnd3, also known as RhoE, is a small GTP-binding protein of the Rho family [16]. Despite the nucleotide-binding capacity of GTPase structure, Rnd3 lacks GTPase activity and cannot hydrolyze GTP-bound nucleotide [17]. Rnd3, a regulator famous for its significant influence on cell shape and migration, is characterized by its unique molecular properties that govern cytoskeletal dynamics [17]. This feature is crucial for the maintenance of structural integrity and functional capacity of cardiomyocytes under physiological conditions [18, 19]. Previous studies have underscored the pivotal role of Rnd3 in the modulation of various cardiovascular conditions, including myocardial infarction, diabetic cardiomyopathy, and atherosclerosis [18, 20, 21]. The observed decrease in Rnd3 expression has been correlated with detrimental impacts on cardiac function, prompting further exploration into its mechanism regarding mitigation of DIC. Recent studies indicated that Rnd3 guarantees mitochondrial integrity by modulating mitochondrial respiration and altering mitochondria oxidative metabolism, thereby mitigating cell death [22, 23]. However, whether Rnd3 influences mitochondrial function in response to Dox stimulation remains to be further elucidated.

In the present study, we demonstrated an evident cardioprotective effect of Rnd3 in DIC. Furthermore, Rnd3 significantly impeded Drp1-induced mitochondrial fission, thereby mitigating Dox-induced PANoptosis. Mechanistically, Rnd3 directly interacts with Rock1 in cytoplasm, which in turn inhibits Drp1 phosphorylation at Ser616, and consequently inhibits mitochondrial fission. By focusing on the Rnd3/Rock1/Drp1 signaling pathway, the present results helped to put forward novel insights with regard to the molecular mechanisms of DIC.

## Methods

### Ethics statement

All experimental animal procedures were conducted in accordance with the Guideline for the Care and Use of Laboratory Animals, published by the US National Institutes of Health (NIH Publication, 8th Edition, 2011), and adhered to the ethical guidelines set forth by the Ethics Committee of the Fourth Military Medical University (Approval ID: 20220617). In brief, mice were housed in a controlled environment with a 12-hour light/12-hour dark cycle with access to food and water ad libitum. Male mice were used. Mice were randomly assigned to either the control group or the experimental group using a random number generator to ensure unbiased allocation of animals to different experimental conditions.

### Experimental animal models

Rnd3^LSP/LSP^ mice (N-Tg) were generated on a C57BL/6 J genetic background using CRISPR/Cas9 technology. In brief, cardiomyocyte-specific Rnd3 transgenic mice (Rnd3-Tg) were created by crossing Rnd3^LSP/LSP^ mice with Myh6-Cre mice. Dox (5 mg/kg/week, TargetMol, T1020, BSN, USA) dissolved in saline (0.9% sterile sodium chloride) was intraperitoneally administered to 8-week-old mice for 4 consecutive weeks. The cumulative dosage of Dox administered to each mouse was 20 mg/kg. The control group was given the same volume of saline vehicles. In the fourth week, mice were anesthetized by 2% isoflurane inhalation in oxygen and sacrificed via cervical dislocation. A schematic diagram of transgenic mice and the information on the primers used for genotyping were provided in Supplementary Informations (Supplementary Information 1: Fig. S1 and Table S1). In order to inhibit Rock1 expression, mice were intraperitoneal injected with saline or Fasudil (10 mg/kg/day, TargetMol, T1606, BSN, USA, dissolved in saline) once daily, which was continued for 6 days. The control group was given the same volume of saline vehicles [24].

### Echocardiography

Echocardiography was employed to assess cardiac function following the final Dox administration. The procedure was conducted using a two-dimension guided M-mode echocardiography system (Visual Sonics; Vevo2100, ON, Canada). Prior to the examination, the chest and upper abdomen of mice were depilated to ensure clear visualization of the heart. During the procedure, mice were continuously anesthetized with 2% isoflurane inhalation while being placed on a heating plate to maintain a constant body temperature at 37 °C. Parameters of cardiac function and ventricular volume were calculated using Vevo computer algorithms, including left ventricular ejection fraction (LVEF) and left ventricular fractional shortening (LVFS).

### CK-MB and LDH measurement

Cardiac injury markers were detected by ELISA according to the manufacturer’s instructions. The lactate dehydrogenase assay kit (Jiancheng Bio, A020-1-2, Nanjing, China) was used to measure the serum lactate dehydrogenase (LDH) level. The creatine kinase MB isoenzyme assay kit (Jiancheng Bio, H197-1-1, Nanjing, China) was used to measure the serum creatine kinase MB isoenzyme (CK-MB) level.

### Histology

Mice were anesthetized with 2% isoflurane inhalation. The chest was depilated to thoroughly expose the heart. The precooled phosphate-buffered saline (PBS) was perfused onto the fresh hearts of mice via the aorta until completely dumped blood in the heart chambers. Subsequently, cardiac tissues were fixed overnight with 4% paraformaldehyde, embedded in paraffin, and cut into 5 μm slices. Masson trichrome staining (Servicebio, G1006, Wuhan, China) was performed to determine myocardial interstitial fibrosis. Fluorescein isothiocyanate (FITC)-labeled wheat germ agglutinin (WGA) staining (Servicebio, L4895, Wuhan, China) was performed to determine the cross-sectional areas of cardiomyocytes.

### Immunofluorescence and Immunohistochemistry

Cardiomyocytes or heart tissues were fixed with 4% paraformaldehyde for 15 minutes, followed by incubation with a permeabilization solution containing Triton X-100 (Beyotime, P0096, Shanghai, China) for 20 minutes at room temperature. Blocking was achieved using goat serum (Beyotime, C0265, Shanghai, China) for 1 hour at room temperature. Cardiomyocytes were incubated with the primary antibody overnight at 4 °C and incubated with a fluorescent secondary antibody for 1 hour at room temperature, and nuclei were stained with DAPI (Beyotime, C1006, Shanghai, China). Images were captured using a confocal fluorescence microscope. For immunohistochemical analysis, slides were first fixed in 4 °C acetone for 10 minutes and then rehydrated in PBS. Blocking was done with 3% BSA for 1 hour, followed by overnight incubation with the primary antibody at 4 °C. Following washing, the sections were labeled with horseradish peroxidase (HRP), stained with diaminobenzidine (DAB), counterstained with hematoxylin, and subsequently examined under a light microscope. Antibody information was shown in Supplementary Informations (Supplementary Information 1: Table S2).

### Transmission electron microscopy (TEM)

Left ventricular heart tissues were minced into approximately 1mm^3^ cubes and were rapidly fixed in 2.5% glutaraldehyde solution (Servicebio, G1101-3ML, Wuhan, China) overnight at 4 °C. Samples were then fixed, washed, dehydrated, embedded, and cut into 60-80 nm ultrathin sections for imaging. Mitochondrial ultrastructure was analyzed by transmission electron microscope (HITACHI, HT7700, Tokoyo, Japan) at 300 kV.

### Adeno-associated virus 9 and viral delivery protocol

To specifically knockdown Rock1 in hearts, the adeno-associated virus 9 (AAV9) vector (Hanbio, Shanghai, China) containing shRNA-Rock1 and GFP fluorescence sequence was used in vivo experiments. The AAV9-shRNA-Rock1 (Hanbio, Shanghai, China) was 5’-GCAGTGTCTCAAATT GAGAAA-3’. Meantime, AAV9-shRNA-Rock1 and AAV9-shRNA-Control were randomly injected into the hearts of Rnd3-Tg and N-Tg through intramyocardial injection. Immunofluorescence staining was employed to detect transfection efficiency after 48 hours, and Western blot was performed to assess the silencing efficacy of Rock1. The AAV9-Drp1 S616D was used to mimic the phosphorylation site of Drp1 ser616 by intramyocardial injection (Hanbio, Shanghai, China). The AAV9-Drp1 S616D was 5’-CCAATTATGCCAGCAGATCCACAGAAAGG-3’. Western blot was performed to assess the efficacy of AAV9-Drp1 S616D 48 hours after transfection.

### Cell culture and treatment

For isolation of cardiomyocytes from 1-3 day-old neonatal mouse hearts, ventricles were excised and dissected into small cubes of approximately 1-2 mm³ using scissors. The cubes were then subjected to sequential digestion with 0.1% collagenase II (Biosharp, BS164-100mg, Hefei, China) to dissociate cardiomyocytes. On one hand, the resulting cell suspension was cultured in a Dulbecco’s Modified Eagle Medium (DMEM) containing 20% fetal bovine serum (FBS) and 1% penicillin-streptomycin for 2 hours at 37 °C in a humidified atmosphere with 5% CO_2_. This step allowed for the separation of neonatal mouse fibroblasts from the neonatal mouse cardiomyocytes. Subsequently, cardiomyocytes were collected and cultured in DMEM supplemented with 20% FBS and 1% penicillin-streptomycin. On the other hand, neonatal mouse fibroblasts were cultured in DMEM supplemented with 10% FBS and 1% penicillin-streptomycin. To identify the role of Rnd3 in DIC, we specifically overexpressed Rnd3 in cardiomyocytes by delivering adenoviruses harboring Rnd3 (Ad-Rnd3) (Hanbio, Shanghai, China) at varying multiplicities of infection (MOIs). The cardiomyocytes were exposed to Dox (2 μM) or PBS medium for an additional 24 hours after adenoviral transfection.

For drug administration, mitochondrial fission inhibitor Mdivi-1 purchased from Sigma Aldrich (Sigma Aldrich, sc-215291B, St. Louis, MO, USA) was used. In this study, a concentration of 50 μM was used as the treatment, while an equal volume of DMSO served as the control. Rock1 inhibition was induced by Fasudil (100μmol/L) injection as described previously [25].

### Mitochondrial isolation

Mitochondria were isolated from cardiomyocytes using cell mitochondria isolation kit (Beyotime, C3606, Shanghai, China), according to the manufacturer’s instructions. The mitochondria and cytoplasm were isolated through differential centrifugation. Briefly, cells were resuspened in a mitochondria extraction reagent (provided in the kit) and homogenized with a microhomogenizer, then placed in ice bath for 15 min. The homogenates were centrifuged at 600 g for 10 min at 4 °C. The supernatants were collected and further centrifuged at 11,000 g for 10 min at 4 °C. The cytosol fraction was collected from the supernatant, And the mitochondrial fraction was collected from the precipitates.

### Analysis of mitochondrial morphology

Mitochondrial morphology was assessed in cardiomyocytes using Mito Tracker Red (Invitrogen, M22426, Carlsbad, CA, USA) staining according to the manufacturer’s instructions. Briefly, cardiomyocytes were stained with Mito Tracker Red (200 nmol/L) for 30 minutes at 37 °C. Images were collected using a confocal fluorescence microscope, and quantitative mitochondrial morphology analysis was performed through image processing using the manual tracker plugin in ImageJ.

### Assessment of mitochondrial function

Mitochondrial ROS (mtROS) production in cardiomyocytes was determined utilizing the Mito-SOX Red mitochondrial superoxide indicator (Invitrogen, M36008, Carlsbad, CA, USA) according to the manufacturer’s instructions. JC-1 assay kits (Beyotime, C2003S, Shanghai, China) were used in cardiomyocytes according to the manufacturer’s instructions to measure mitochondrial membrane potential (MMP). Under normal ΔψM conditions, JC-1 forms aggregates to exhibit a red appearance (JC-1 aggregates). However, when ΔψM collapsed, the dye transitioned to a green appearance (JC-1 monomers). Thus, a reduction in the red/green ratio exhibited a decrease in MMP and impairment of mitochondrial function. Images were captured using a confocal fluorescence microscope.

### Assay of cells undergoing apoptosis, necroptosis, and pyroptosis

Dual staining with YO-PRO-1 (YP1) and propidium iodide (PI) was employed to detect cell death using Apoptosis and Necrosis Detection Kit (Beyotime, C1075S, Shanghai, China) according to the manufacturer’s instructions. Briefly, cardiomyocytes were cultured in 24-well plates and subsequently treated with YP1 and PI reagents. Stained cells were subsequently analyzed under a fluorescence microscope. YP1-positive cells, exhibiting a green fluorescence, indicated cells undergoing apoptosis or necroptosis, whereas PI-positive cells, displaying a red fluorescence, denoted cells undergoing necroptosis or pyroptosis.

### CCK-8 viability

Cell viability was determined via the Cell Counting Kit-8 (Sigma Aldrich, 96992, St. Louis, MO, USA). Briefly, cardiomyocytes were plated in 96-well plates and treated with Dox. According to the manufacturer’s instructions, the absorbance at 450 nm was read using the microplate reader (Bio‐Rad, Hercules, CA, USA).

### Analysis of bulk RNA-seq data

For in vitro gene delivery, Ad-Rnd3 was employed to upregulate Rnd3 expression. Following transduction for 48 hours, cardiomyocytes were treated with 2 μM Dox for 24 hours. Total RNA was extracted from the cardiomyocytes using a TRIzol reagent (Invitrogen, 15596018, Carlsbad, CA, USA). The RNA sequencing was performed by a commercial vendor (Personalbio, Shanghai, China). After obtaining the raw sequencing data, the read counts were normalized and further processed in R ClusterProfiler package. Kyoto Encyclopedia of Genes and Genomes (KEGG) annotated the function of differentially expressed genes. And Gene set enrichment analysis (GSEA) was conducted using the MSigDB (Supplementary Information 3 and Supplementary Information 4).

### Protein-protein docking

The potential binding mode of Rnd3 and Rock1 was predicted by Rigid protein-protein docking (HDOCK and Cluspro). The PDB format of the protein structural domains of Rnd3 and Rock1 was downloaded from the Protein Data Bank database. The docking sites and the calculation of the docked binding energy were used by the Pymol 2.3.0 program.

### Co-immunoprecipitation

Co-immunoprecipitation (Co-IP) was performed using an IP/Co-IP Kit (Thermo Fisher Scientific, 88804, Waltham, MA, USA) according to the manufacturer’s instructions. Initially, cellular lysates containing 500 μg of protein were mixed with 5 μg of primary antibody or isotype control immunoglobulin G per sample incubated overnight at 4 °C. Then, protein A/G magnetic beads were mixed with the antigen/antibody complex for 1 hour at 37 °C. The beads were washed twice with IP Lysis/Wash Buffer and then once with purified water. Finally, the protein was collected and analyzed by Western blot. Antibody information was shown in Supplementary materials (Supplementary Information 1: Table S2).

### Small interfering RNA (siRNA) transfection

The siRNA procured from Hanbio (Hanbio, Shanghai, China) was employed to achieve the knockdown of Rock1 in cardiomyocytes. The targeting sequences of siRNA-Rock1 were 5’-GAAGAAACATTCCCTATTC-3’. Cardiomyocytes were transfected with siRNA-Rock1 using Lipofectamine 2000 (Thermo Fisher Scientific, 11668500, Waltham, MA, USA) according to the manufacturer’s instructions. Briefly, the diluted siRNA and Lipofectamine 2000 were mixed and incubated for 20 minutes at room temperature and were incubated with cells for 24 hours for further study. Western blot and PCR were performed to assess the silencing efficacy of Rock1.

### Quantitative real-time PCR

The total RNA was extracted from heart tissue or cardiomyocytes utilizing TRIzol™ reagent (Invitrogen, 15596018, Carlsbad, CA, USA). The RNA concentration was estimated by the NanoDrop™ One/OneC nucleic acid (Thermo Fisher Scientific, 701-058108, USA). cDNA was synthesized utilizing the PrimeScript™ RT reagent Kit and gDNA Eraser (Takara, RR047A, Tokyo, Japan). Quantitative analysis was performed using TB Green® Premix Ex Taq™ II (Takara, RR820A, Tokyo, Japan) and StepOnePlusTM RealTime PCR (Thermo Fisher Scientific, 43766, Waltham, MA, USA). Specific primers sequence was listed in Supplementary materials (Supplementary Information 1: Table S3).

### Western blot

Proteins were isolated from tissue or cells by lysing them using RIPA lysis buffer (Beyotime, P0013E, Shanghai, China), which contained protease and phosphatase inhibitors. Protein concentration was determined using the BCA assay (Beyotime, P0010, Shanghai, China). Proteins were added into 10% or 12% SDS-PAGE gels for electrophoresis and then transferred onto PVDF membranes with a 0.22-μm pore size (Millipore, ISEQ00010, Billerica, MA, USA). The membranes were blocked in 5% skim milk for 1 hour at 37 °C, and incubated with the specific primary antibody at 4 °C overnight. Then the membranes were incubated with isotype-matched secondary antibodies for 1 hour at 37 °C, and the specific antigen-antibody complexes were detected by using a chemiluminescence system (Amersham Bioscience, Buckinghamshire, UK). Antibody information was shown in Supplementary materials (Supplementary Information 1: Table S2).

### Co-localization analysis

The co-localization of protein signals was analyzed using the Plot Profile plugin in ImageJ software. A linear selection was drawn across the regions of interest, and the default settings were applied for signal analysis. The data from each channel were documented, and the changes in intensity along the selected line were quantified using GraphPad Prism 9.0.0.

### Statistical analysis

All experimental data were presented as mean ± standard deviation (SD) and were analyzed using GraphPad Prism 9.0.0 software. To estimate differences between the two groups, a two-tailed unpaired Student’s t-test was employed. For multi-group comparisons, a one-way analysis of variance (ANOVA) with the Bonferroni post-hoc analysis was conducted. A p-value less than 0.05 was deemed statistically significant, indicating a meaningful difference among compared groups. The number of biological replicates is denoted by n, as specified in the corresponding figure legends.

## Results

### Rnd3 expression is markedly downregulated in DIC

**Fig. 1:**
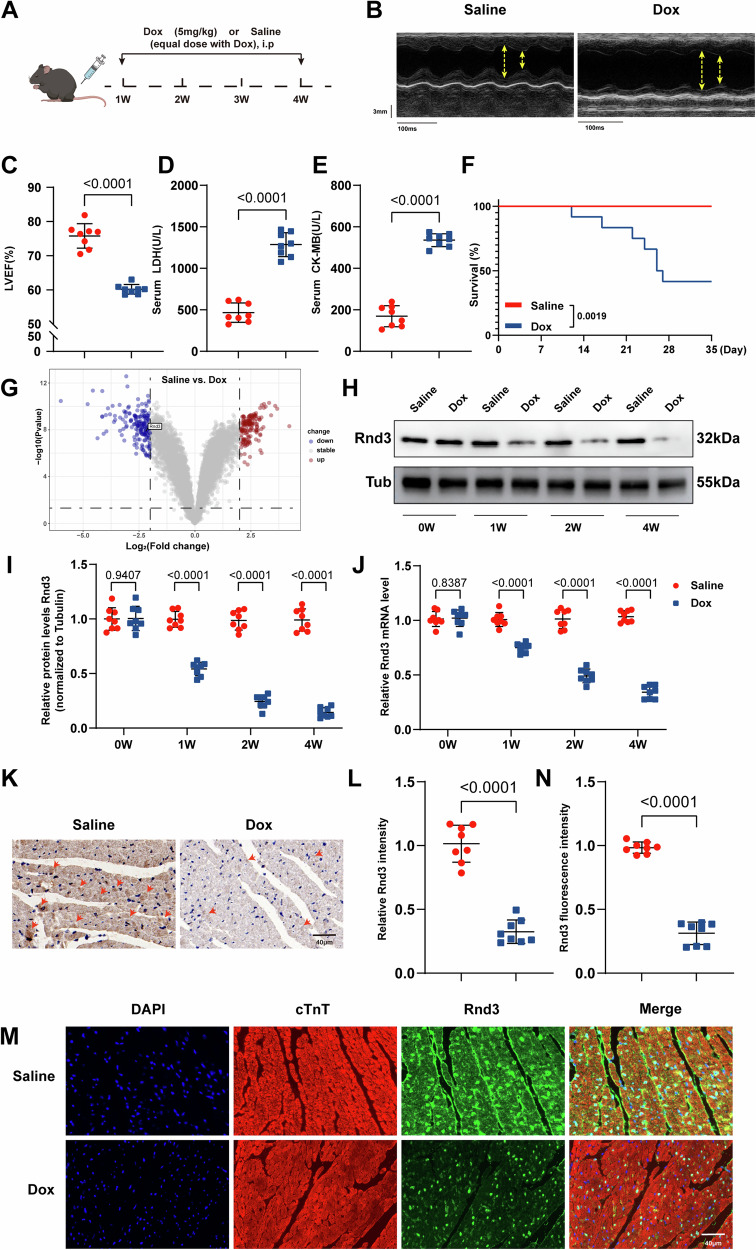
Rnd3 expression was markedly downregulated in DIC. **A** Schematic diagram depicting the experimental strategy. **B** Representative M-mode echocardiographic images of the left ventricle in mice received Saline or Dox. **C** Left ventricular ejection fraction (LVEF) assessed by echocardiography (*n* = 12). **D**, **E** The serum levels of cardiac LDH and CK-MB were significantly elevated in the Dox-treated WT mice compared to the WT mice using ELISA (*n* = 8). **F** Kaplan-Meier survival analysis showing deceased survival in Dox-treated WT mice compared with WT mice (*n* = 12). **G** Volcano plot for differential gene expression in the Dox-treated WT mice compared to the WT mice, *P*-value < 0.05. **H** Cardiac Rnd3 protein level was evaluated from the indicated treatment groups at 0,1, 2 and 4 weeks following Dox treatment. **I** Quantitative analysis of Rnd3 protein expression (*n* = 8). **J** Quantitative analysis of Rnd3 mRNA expression (*n* = 8). **K** Representative images of immunohistochemical staining of Rnd3 from mice heart in control groups and Dox groups (yellow arrow), Scale bar = 40 μm. **L** Quantitation of immunohistochemical staining of the Rnd3-positive area of (**K**) (*n* = 8). **M** Representative images of immunofluorescence staining of Rnd3 (green) in cardiomyocytes (red), Scale bar = 40 μm. cTnT were employed as markers for cardiomyocytes. **N** Quantitative analysis of mean Rnd3 fluorescence intensity (*n* = 8). Data were presented as mean ± SD. Student’s t-test was used for statistical analysis in (**C**)–(**F**), (**I**), (**J**), (**L**), and (**N**).

To discern possible factors contributing to DIC, a rodent model was established using intraperitoneal (i.p.) injections of Dox (5 mg/kg, four doses, once per week) for 4 weeks (Fig. 1A). In agreement with previous studies [26], Dox challenge significantly reinforced the reduction in left ventricular ejection fraction (LVEF) (Fig. 1B and C) and the increase in LDH and CK-MB levels (Fig. 1D and E), denoting successful establishment of the DIC model in vivo. Cumulative survival was monitored in mice, and the result indicated that the survival rate was overtly reduced in the Dox group compared with the control group (P < 0.01) (Fig. 1F). We then analyzed the gene expression profiles (GSE213983) of left ventricle tissues from Dox or Saline-treated mice to identify the potential key regulators of DIC (Supplementary Information 2). The expression pattern of key factors involved in DIC was displayed in the Volcano plot (Fig. 1G). The present analysis indicated a prominent downregulation of Rnd3 expression in the Dox group as compared with the control group. Hence, we hypothesized that Rnd3 may serve as a critical pathogenic factor in DIC.

To verify our hypothesis, we next assessed Rnd3 expression in cardiac tissue. The present results displayed a noteworthy decline of Rnd3 in protein levels and mRNA levels in the Dox group compared to the control group (Fig. 1H–J). Immunohistochemistry also indicated Rnd3 reduction following Dox challenge (Fig. 1K, L). Consistently, compared with their respective controls, decreased expression of Rnd3 in hearts from Dox-treated mice was further consolidated by immunofluorescence staining (Fig. 1M, N). Collectively, these results strongly suggested that Rnd3 plays an important regulatory role in DIC.

### Upregulation of Rnd3 in cardiomyocytes ameliorates Dox-induced cardiac dysfunction and remodeling in mice

**Fig. 2:**
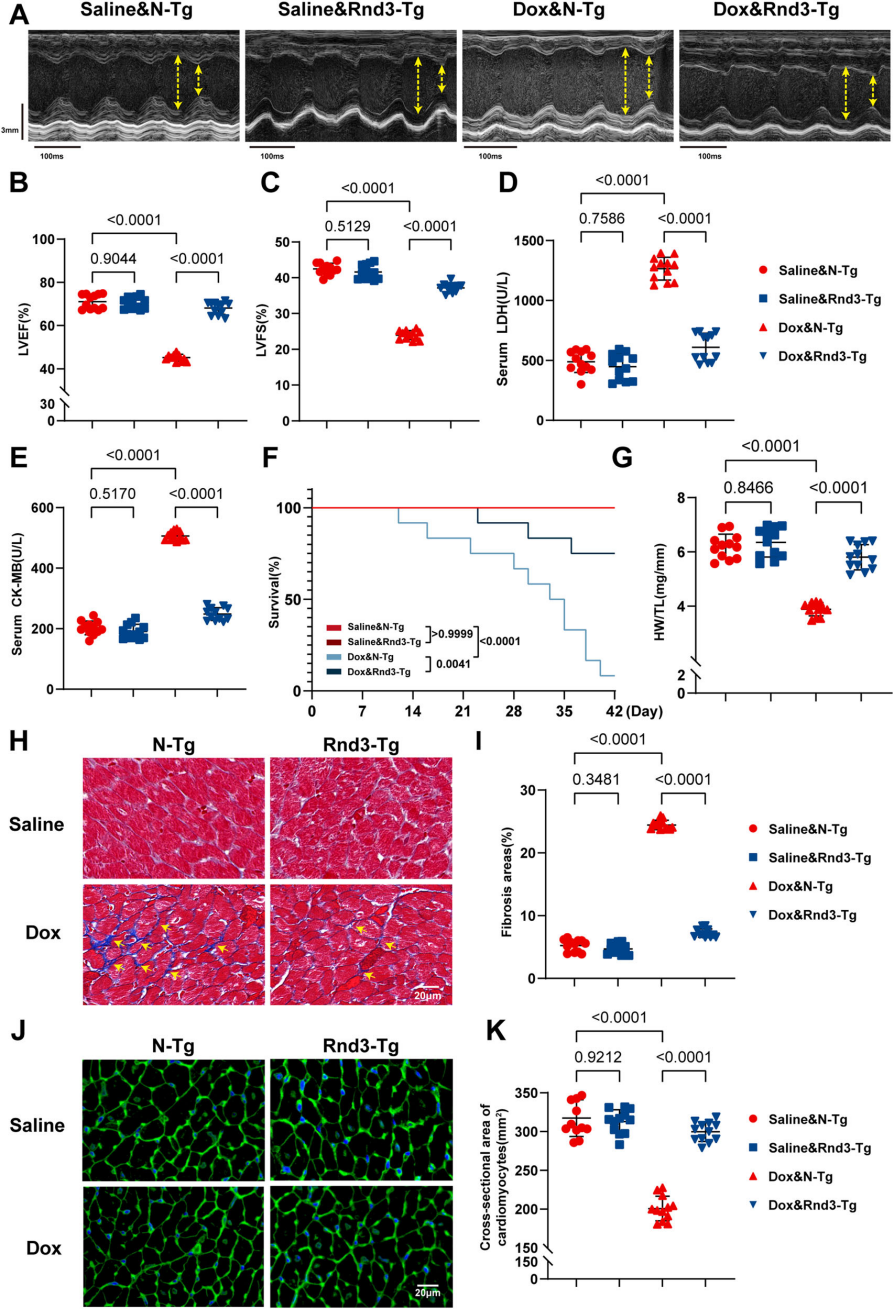
Cardiomyocyte-specific Rnd3 overexpression ameliorated Dox-induced cardiac dysfunction and remodeling in mice. **A** Representative M-mode echocardiographic imaging of different treatment mice heart. **B**, **C** LVEF and LVFS assessment by echocardiography (*n* = 12). **D**, **E** Serum level of LDH and CK-MB measured using ELISA (*n* = 12). **F** Survival curve of mice during the experimental period (*n* = 12). **G** Statistical analysis of heart weight (mg) and tibia length (mm) ratio (HW/TL) (*n* = 12). **H**, **I** Representative Masson trichrome staining in the interstitial and quantification of the LV collagen volume (yellow arrow) (*n* = 12), scale bar = 20 μm. **J**, **K** Representative images of WGA-stained sections, and quantification of cardiomyocytes cross-sectional area based on WGA staining (*n* = 12), scale bar = 20 μm. Data were presented as mean ± SD. One-way ANOVA was used for statistical analysis in (**B**)–(**G**), (**I**), and (**K**).

To further analyze the function of Rnd3 in vivo, cardiomyocyte-specific Rnd3 transgenic mice were generated by crossbreeding Rnd3^LSP/LSP^ mice with Myh6-Cre mice. Mice were intraperitoneally injected with Dox or Saline for 4 weeks. Echocardiography was performed 1 week after the final dose of Dox injection to assess cardiac function following the Dox challenge, and the results revealed a profound systolic dysfunction, as indicated by LVEF and LVFS. Notably, cardiomyocyte-specific Rnd3 overexpression ameliorated Dox-evoked systolic dysfunction, evidenced by increased LVEF and LVFS (Fig. 2A–C). In addition, Rnd3 overexpression reversed the elevated levels of serum cardiac enzymes LDH and CK-MB (Fig. 2D, E), and overtly improved the survival rate in the realm of DIC (Fig. 2F). Of note, compared with the N-Tg mice, restricted mean survival time (RMST) was significantly increased in Rnd3-Tg mice following Dox treatment (37.42 d vs 30.58 d, p <0.05) (Supplementary Information 1: Fig. S2). Furthermore, the present findings revealed that Rnd3 overexpression led to an elevation in heart weight related to tibial length ratio (HW/TL) (Fig. 2G). Moreover, we performed masson trichrome staining to assess cardiac fibrosis, and the results exhibited notably elevated fibrosis in response to Dox insult. However, compared with the N-Tg group, the Rnd3-Tg group exhibited a remarkable reduction in cardiac fibrosis following Dox administration (Fig. 2H and I). In alliance with the present observations, WGA staining indicated that Rnd3 overexpression markedly attenuated Dox-induced cardiomyocyte atrophy as evidenced by increased cross-sectional area (Fig. 2J, K). Collectively, these results strongly suggested that Rnd3 upregulation significantly improved cardiac dysfunction and remodeling in DIC.

### Restoration of Cardiac Rnd3 mitigates Dox-induced mitochondrial dysfunction

**Fig. 3:**
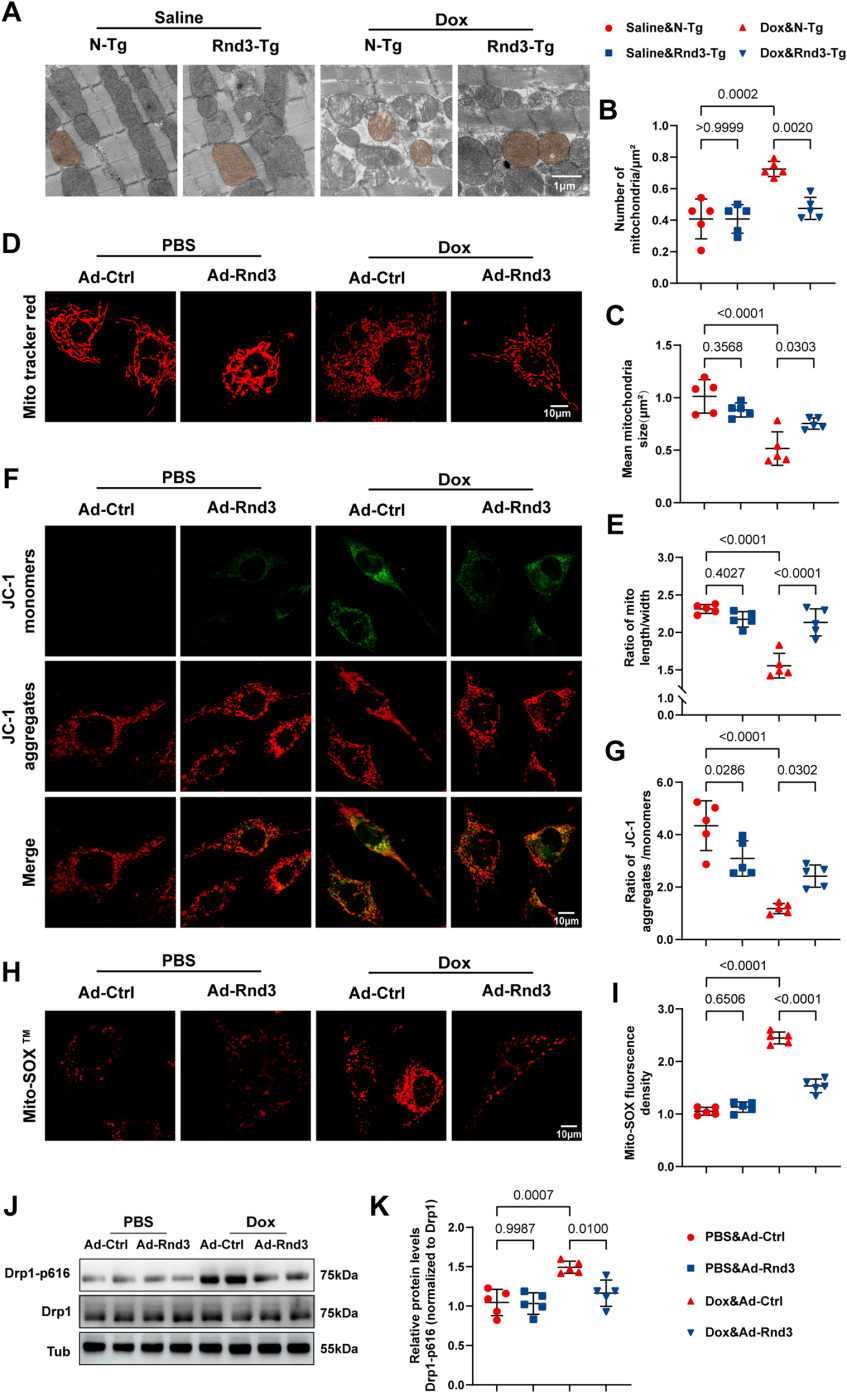
Rnd3 overexpression mitigated Dox-induced mitochondrial dysfunction. **A** Representative transmission electron microscope images show the damaged mitochondrial with loss of cristae in cardiomyocytes, scale bar = 10 μm. **B**, **C** Quantitative analysis of mitochondrial number per field and mean mitochondrial size (*n* = 5). **D** Representative confocal images of Mitochondrial Tracker Red in PBS or Dox-treated cardiomyocytes with Ad-Control or Ad-Rnd3, scale bar = 10 μm. **E** Quantitative analysis of mitochondrial morphology (*n* = 5). **F** Representative confocal microscopic images of JC-1 staining. Red fluorescence represents JC-1 aggregate and green fluorescence denotes JC-1 monomer, scale bar = 10 μm. **G** Quantitative analysis of the MMP assessed through JC-1 red/green fluorescence intensity (*n* = 5). **H** Representative immunofluorescence images of Mito-SOX staining, scale bar = 10 μm. **I** Quantitative analysis of mitochondria-derived superoxide production in cardiomyocytes (*n* = 5). **J** Representative Western blot images of mitochondrial dynamics-related proteins including Drp1-p616 and Drp1 in cardiomyocytes from Ad-Control or Ad-Rnd3 group. **K** Quantitative analysis of Drp1-p616/Drp1 protein expression (*n* = 5). Data were presented as mean ± SD. One-way ANOVA was used for statistical analysis in (**B**), (**C**), (**E**), (**G**), (**I**), and (**K**).

Modulation of mitochondrial dysfunction represents a supplemental molecular mechanism to counteract DIC [27]. To determine whether Rnd3 is involved in the regulation of mitochondrial dysfunction in DIC, mitochondrial morphology was assessed using transmission electron microscopy. As shown in Fig. 3A, abnormal mitochondria were observed in Dox-challenged cardaic tissues (including profound damage to mitochondrial cristae). Quantification analysis of mitochondrial morphology showed smaller mean mitochondrial size and increased numbers of mitochondria in the Dox group as compared to the Saline group (Fig. 3B, C). In contrast, Rnd3 overexpression decreased the quantity of small and circular mitochondria in myocardial tissues following Dox administration (Fig. 3B, C). To further elucidate the role of Rnd3 in Dox-induced mitochondrial injury, cardiomyocytes transfected with Ad-Rnd3 or Ad-Control were incubated in vitro with Dox or PBS medium for 24 hours to mimic DIC. We first assessed the Rnd3 expression at various MOI of Ad-Rnd3 using Western blot analysis. The results demonstrated that the expression levels of Rnd3 were significantly upregulated following Ad-Rnd3 transfection, with peak expression observed at an MOI of 50 (Supplementary Information 1: Fig. S3A, B). This MOI was chosen for subsequent experiments. Next, we measured cell viability through the CCK-8 assay and noted that Dox reduced cardiomyocyte viability in a concentration-dependent manner. Notably, Dox at the concentration of 2 μM evoked moderate cardiomyocyte injury, with cell viability reduced by about 50% (Supplementary Information 1: Fig. S4). Consistent with TEM observations in cardiac tissues, Mito Tracker staining in cardiomyocytes supported the finding that Dox-induced mitochondrial fragmentation was alleviated by Rnd3 overexpression (Fig. 3D and E). We further assessed the effects of Rnd3 on mitochondrial function through mitochondrial membrane potential (MMP) and mitochondrial reactive oxygen species (mtROS). JC-1 staining revealed a significant shift from red to green fluorescence in Dox-exposed cardiomyocytes, indicating collapsed MMP. However, this impact was effectively ameliorated by Rnd3 overexpression (Fig. 3F, G). Additionally, utilizing Mito-SOX Red staining together with a confocal fluorescence microscope, we observed an increased fluorescence intensity of mtROS in the Dox group, compared to the control group (Fig. 3H, I). Therefore, the present results confirmed that replenishment of Rnd3 inhibited mitochondrial dysfunction caused by Dox treatment. Multiple studies have documented that Drp1, a critical regulator of mitochondrial fission, is indispensable for the modulation of mitochondrial dynamics, which is pivotal in governing the size and mass of mitochondria [8, 28, 29]. Accumulative evidence indicated that Drp1 Ser616 phosphorylation plays a pivotal role in recruiting Drp1 from cytoplasm to mitochondria, and subsequently facilitates mitochondrial fragmentation [30, 31]. To explore whether Rnd3 participates in mitochondrial fission through Drp1 phosphorylation, we conducted a detailed analysis of Drp1 phosphorylation levels in cardiomyocytes (Fig. 3J). The results indicated that Rnd3 overexpression markedly attenuates the phosphorylation of Drp1 specifically at the Ser616 (Fig. 3K).

To further identify the role of the phosphorylation of Drp1 at Ser616 in DIC, an adeno-associated virus 9 expressing Drp1 S616D (AAV9-Drp1 S616D) was constructed to mimic phosphorylation of Drp1 at Ser616. Subsequently, we specifically overexpressed a Drp1 phosphomimetic (Drp1 S616D) in cardiomyocytes by delivering AAV9-Drp1 S616D into N-Tg mice or Rnd3-Tg mice through intramyocardial injection. The efficiency of Drp1 S616D overexpression in myocardial tissues was verified using Western blot (Supplementary Information 1: Fig. S5A and B). Echocardiography revealed that AAV9-Drp1 S616D injection reversed Rnd3-mediated cardioprotection following Dox treatment, characterized by increased LVEF and LVFS (Supplementary Information 1: Fig. S5C-E). Meanwhile, transmission electron microscopy of cardiomyocyte mitochondria showed that Drp1 S616D overexpression offset Rnd3-offered protective effects on mitochondrial injury (Supplementary Information 1: Fig. S5F-H). These findings collectively suggested that Rnd3 serves as a protective factor against Dox-induced mitochondrial fission by inhibiting Drp1 phosphorylation at Ser616.

### Overexpression of Rnd3 in cardiomyocytes reverses Dox-induced apoptosis, necroptosis, and pyroptosis (PANoptosis)

**Fig. 4:**
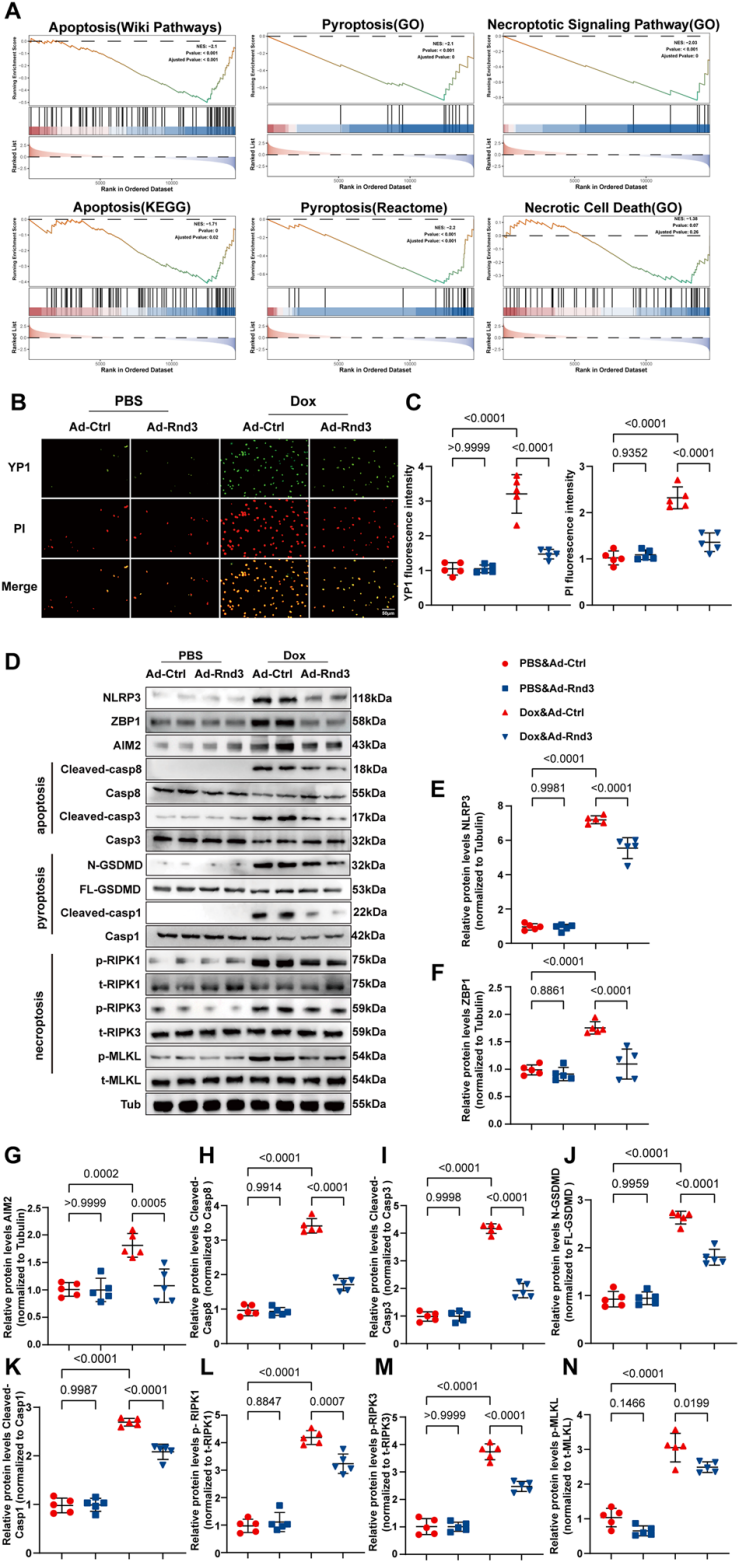
Rnd3 overexpression reversed Dox-induced cardiomyocyte apoptosis, necroptosis, and pyroptosis (PANoptosis). **A** Gene set enrichment analysis, based on Wiki pathways, GO, KEGG and REACTOME, showing apoptosis, pyroptosis, and necroptosis signaling pathways (PANoptosis). **B** Representative immunofluorescence images showing YP1-positive cells (green) which may undergo apoptosis or necroptosis and PI-positive cells (red) which may undergo necroptosis, or pyroptosis, scale bar = 50 μm. **C** Quantitative analysis of YP1 and PI-positive cells (*n* = 5). **D** Representative Western blot images of NRLP3, ZBP1, AIM2, Cleaved-casp8, Casp8, Cleaved-casp3, Casp3, N-GSDMD, FL-GSDMD, Cleaved-casp1, Casp1, p-RIPK1, t-RIPK1, p-RIPK3, t-RIPK3, p-MLKL, and t-MLKL protein levels. **E**–**N** Quantitative analysis of NRLP3, ZBP1, AIM2, Cleaved-casp8/Casp8, Cleaved-casp3/Casp3, N-GSDMD/FL-GSDMD, Cleaved-casp1/Casp1, p-RIPK1/t-RIPK1, p-RIPK3/t-RIPK3, and p-MLKL/t-MLKL protein expression (*n* = 5). Data were presented as mean ± SD. One-way ANOVA was used for statistical analysis in (**C**), (**E**), and (**F**)–(**N**).

To gain a deeper understanding of Rnd3-ameliorated cardiac remodeling and dysfunction in DIC, a comprehensive RNA-sequencing analysis on cardiomyocytes transfected with Ad-Rnd3 and Ad-Control was conducted following Dox challenge. Gene set enrichment analysis (GSEA) revealed that apoptosis, necroptosis, and pyroptosis pathways (P adjusted, <0.05) were dramatically suppressed in the Ad-Rnd3 group as compared with the Ad-Control group (Fig. 4A and Supplementary Information 1: Fig. S6). The results indicated that Rnd3 may exert its protective effects in Dox-treated cardiomyocytes through inhibiting these cell death pathways. Previous studies have indicated that apoptosis, necroptosis, and pyroptosis were involved in cardiac remodeling and dysfunction in DIC [32–34]. PANoptosis, a recently discovered type of programmed cell death, is regulated by the PANoptosome which is a complex assembly of multiple proteins that includes sensors including AIM2, ZBP1, and NLRP3 [35]. This intricate structure functions as a dynamic framework, attracting proteins such as GSDMD, Caspase-1, Caspase-3, Caspase-8, MLKL, RIPK1, and RIPK3 to orchestrate the concurrent appearance of PANoptosis [10, 36, 37]. However, the underlying mechanism of PANoptosis in DIC remains mysterious and demands further exploration to be elucidated [11]. To further explore the occurrence of PANoptosis, we performed dual staining with YP1 and PI in cardiomyocytes and found that Dox treatment drastically triggered PANoptosis, including elevated YP-1 positive cells (indicating cellular apoptosis or necroptosis) and PI-positive cells (indicating cellular necroptosis or pyroptosis). Of note, Rnd3 overexpression significantly reduced the number of cell deaths (Fig. 4B, C). Subsequently, the present results indicated that Dox intervention significantly enhanced PANoptosis in cardiomyocytes, as indicated by elevated levels of PANoptosome components such as AIM2, ZBP1, and NLRP3. Furthermore, we observed an increase in the expression levels of activated markers associated with apoptosis (Cleaved-caspase-3 and Cleaved-caspase-8), pyroptosis (N-GSDMD and Cleaved-caspase-1), and necroptosis (p-MLKL, p-RIPK1, and p-RIPK3) in Dox-treated cardiomyocytes. Notably, the present results demonstrated an evident decrease in PANoptosis by Rnd3 upregulation (Fig. 4D–N). Collectively, these results suggested that Rnd3 acts as a cardioprotective role in mitigating Dox-induced PANoptosis.

### Inhibition of mitochondrial fission mitigates Dox-induced PANoptosis

Substantial evidence demonstrated that Drp1 phosphorylation at Ser616 is required for mitochondria fission [38]. The present results revealed that Dox augments Drp1 phosphorylation at Ser616, whereas the upregulation of Rnd3 ameliorated this effect, consequently mitigating mitochondrial fission. To further confirm that Rnd3 mediated cardioprotective effect in the Drp1 phosphorylation-dependent manner, Mdivi-1, an inhibitor of Drp1 at Ser616 [39], was used to assess an association between mitochondrial fission and PANoptosis in Dox-treated cardiomyocytes. Su and associates demonstrated that 50 μM Mdivi-1 could inhibit mitochondrial fission in cardiomyocytes, with appropriate cellular toxicity [40]. Thus, 50 μM Mdivi-1 was used as the effective concentration in this study. We subsequently evaluated the effects of Mdivi-1 through Western blot analysis and the results indicated that the levels of Drp1 phosphorylation were significantly decreased in cardiomyocytes (Supplementary Information 1: Fig. S7A, B). The count of YP1-positive cells and PI-positive cells was significantly decreased by Rnd3 supplementation in cardiomyocytes exposed to Dox insults. However, no significant alterations were found in the PANoptosis of cardiomyocytes with Mdivi-1 treatment in the Ad-Rnd3 group compared to Ad-Control (Supplementary Information 1: Fig. S7C, D). Subsequently, we further examined Dox-induced PANoptosis in cardiomyocytes using Western blot. Consistently, Rnd3 did not exert further protective effects against Dox-induced PANoptosis in the situation of Mdivi-1 treatment (Supplementary Information 1: Fig. S7E–O). These results underscored that Rnd3 mitigated Dox-induced PANoptosis through mediating the phosphorylation of Drp1 at Ser616 and mitochondrial fission.

### Rock1 serves as a downstream regulator and directly binds with Rnd3 in the cytoplasm of cardiomyocytes

**Fig. 5:**
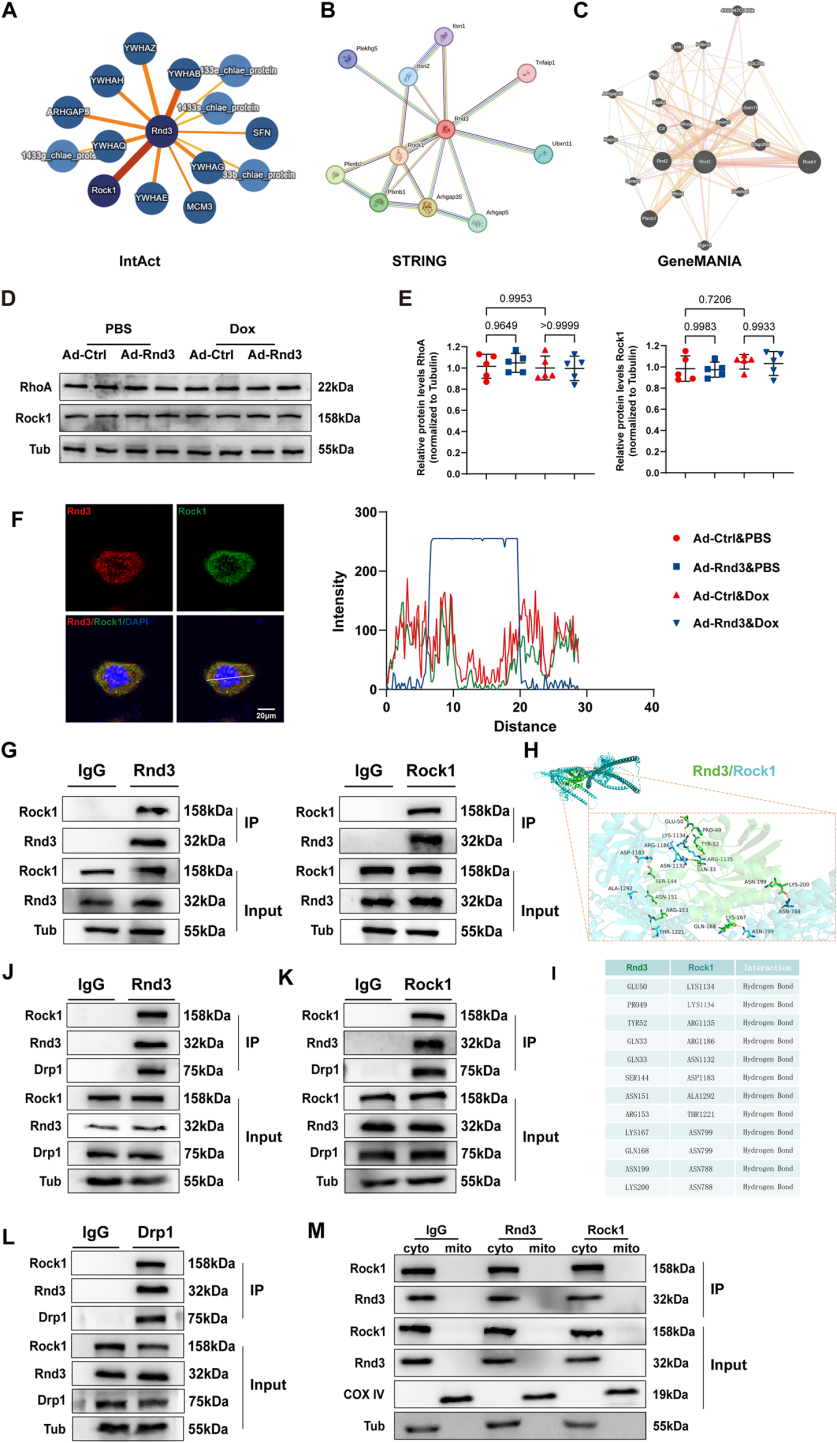
Rock1 played a pivotal role in the Rnd3-induced mitigation of DIC. **A**–**C** IntAct, STRING and GeneMANIA databases indicated Rock1 as a potential interacting protein with Rnd3. **D** Representative Western blot images of RhoA/Rock pathway related protein in cardiomyocytes from Ad-Control or Ad-Rnd3 group. **E** Quantitative analysis of RhoA/Rock pathway related protein expression (*n* = 5). **F** Representative immunofluorescence images of Rnd3 and Rock1 in cardiomyocytes. And quantitative analysis of the colocalization between Rnd3 and Rock1. **G** Cell lysates of primary cardiomyocytes were immunoprecipitated with IgG, Rnd3 or Rock1 antibodies, and Western blot were performed using Rnd3 and Rock1 antibodies. **H** Structure-based protein interaction interface analysis between Rnd3 and Rock1 using the HDOCK server. **I** The docking sites of Rnd3 and Rock1. **J**–**L** Interaction among Rnd3, Rock1 and Drp1 in cardiac tissues was examined by IP-Western blot assay. **M** Interaction among Rnd3, Rock1 in cytoplasm or mitochondrial examined by IP-Western blot assay. Data were presented as mean ± SD. One-way ANOVA was used for statistical analysis in (**E**).

To identify the underlying mechanisms of Rnd3 regulated PANoptosis and mitochondrial fission, we undertook a comprehensive review of various public protein-protein interaction databases (IntAct, STRING, GeneMANIA, and MINT) (Fig. 5A–C and Supplementary Information 1: Figs. S8 and 9). Among these candidates, Rock1 exhibited significant interaction with Rnd3, and this interaction has been recently reported in various pathological conditions [41, 42]. Rock1, known as a serine/threonine kinase, is the downstream signal molecule of RhoA signaling [43]. RhoA/Rock1 signaling pathway is closely involved in the pathological myocardial remodeling [44–46]. We further assessed the activation of RhoA/Rock1 signaling pathway using Western blot analysis. Interestingly, Rnd3 overexpression had no obvious effect on the expression of RhoA and Rock1 in cardiomyocytes under Dox intervention. Based on the present results, we considered that Rnd3 plays an important role in DIC through inhibiting the activity of Rock1 rather than regulating the expression of Rock1 (Fig. 5D, E). To further investigate the association between Rnd3 and Rock1, a double-labeling immunofluorescence staining was performed in vitro and the results showed that Rnd3 and Rock1 mainly colocalized in the cytoplasm (Fig. 5F). Consistent with the double-labeling immunofluorescence staining, co-immunoprecipitation assays exhibited robust binding between Rnd3 with Rock1 (Fig. 5G). We further conducted reverse IP with Rock1 antibodies to confirm the specific interaction between Rnd3 and Rock1 in cardiomyocytes (Fig. 5G). Subsequently, using the HDOCK server, the best docking model was selected based on docking score (docked binding energies: -237.94 kcal/mol) and confidence score (0.8531) (Supplementary Information 1: Table S4). The molecular docking analysis revealed that Rnd3 engages in a stable interaction with Rock1, primarily through hydrophobic interactions mediated by critical amino acid residues (Fig. 5H and I). The hydrogen bonding interaction (117.08 Å) served as a pivotal power in mediating the stable association between Rnd3 and Rock1, especially contributing to their functional interplay. Meantime, Using Cluspro analysis, Rock1 and Rnd3 were selected as the receptor protein and the ligand protein, respectively. The optimal docking configuration was selected based on the cluster size (Supplementary Information 1: Table S5). The molecular docking analysis revealed that Rnd3 also engages in a stable interaction with Rock1, primarily through the hydrogen bonding interaction mediated by critical amino acid residues (Supplementary Information 1: Fig. S10A and B). Previous researches reported that Rnd3 were constructed with domain of N-terminal extensions (Rnd3ΔN, 1-16 amino acid fragment), a core GTP-binding region (Rnd3 Core domain, 16-200 amino acid fragment) and C-terminal extensions (Rnd3ΔC, 200-244 amino acid fragment) [47], and Rock1 were constructed with domain of Rock1 Kinase (1-338 amino acid fragment) and Rock1Δ Kinase (338-1354 amino acid fragment) [48]. Interestingly, the present molecular docking results showed that binding of Rnd3 with Rock1 may depend on the core GTP-binding region of Rnd3 (Rnd3 Core domain, 16-200 amino acid fragment) and Rock1ΔKinase (338-1354 amino acid fragment) (Supplementary Information 1: Fig. S10C). Furthermore, to delve deeper into the functional interplay among Rnd3, Rock1 and Drp1, co-immunoprecipitation assays were conducted in heart tissues. Consistent with our current results, a direct interaction between Rnd3, Rock1 and Drp1 was observed (Fig. 5J-L). Meantime, in order to examine the interacting location of Rnd3 and Rock1, mitochondria was isolated from cardiomyocytes for co-immunoprecipitation assays. The results revealed that the complex of Rnd3 and Rock1 was undetectable, indicating that their combination was specially localized in the cytoplasm (Fig. 5M). Upon the experimental evidence, we concluded that Rock1 occupied a crucial role within the intricate machinery of Rnd3 regulation in mitochondrial fission, ultimately mitigating the progress of DIC. Collectively, Rock1 played a pivotal role in Rnd3-provoked protection against DIC.

### Rnd3 ameliorates Dox-induced mitochondrial fission through Rock1

**Fig. 6:**
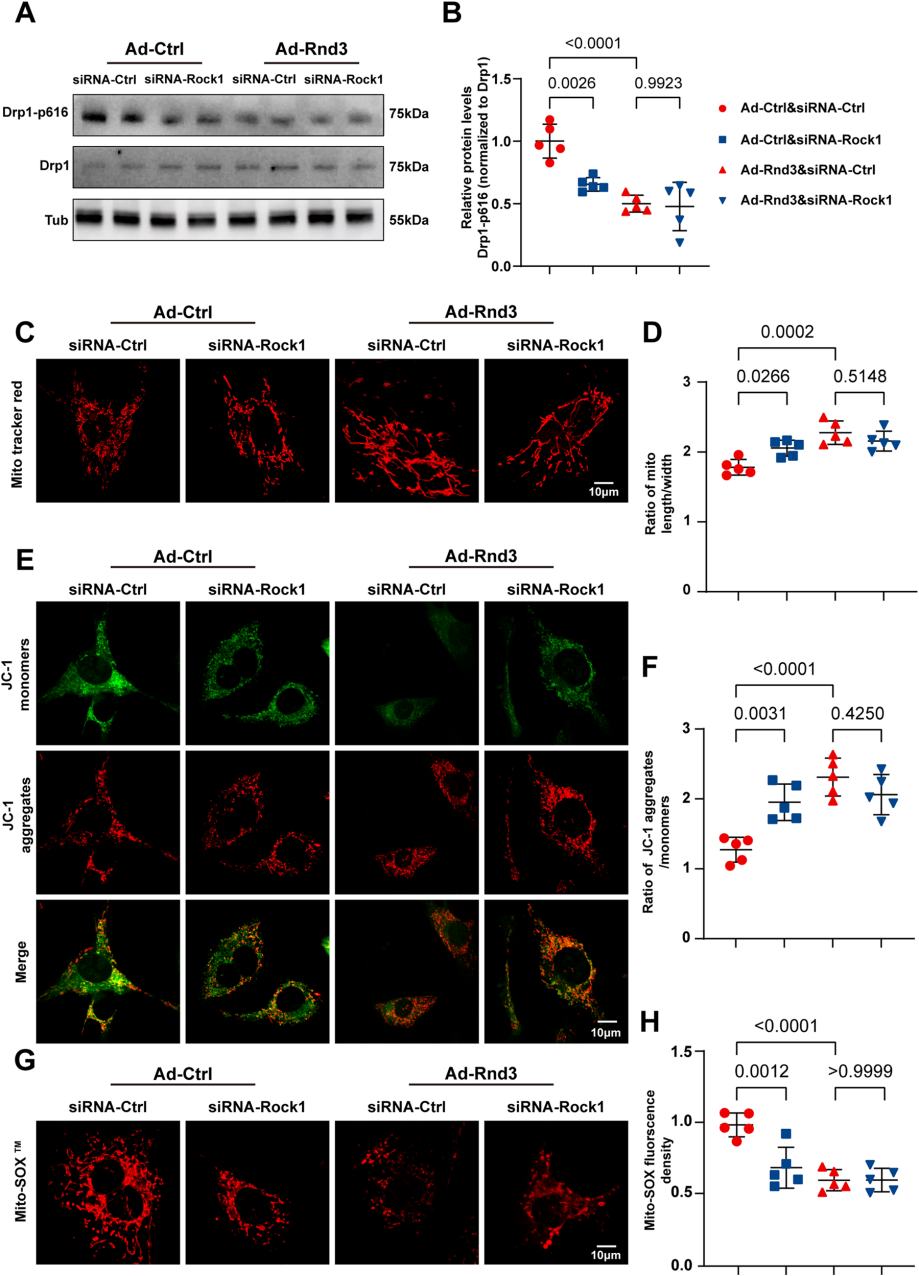
Rnd3 bond to Rock1 to mediate Dox-induced mitochondrial fission. **A** Cardiomyocytes were infected with siRNA-control, siRNA-Rock1, Ad-Control, or Ad-Rnd3, and then treated with Dox (2 μM) for the indicated time periods. Representative Western blot images of Drp1-p616, and total Drp1 protein levels for each group. **B** Quantitative analysis of Drp1-p616/Drp1 protein expression (*n* = 5). **C** Representative mitochondrial images of Mito tracker staining obtained by confocal microscope, scale bar = 10 μm. **D** Quantitative analysis of mitochondrial morphology. **E** Representative immunofluorescence images of JC-1 staining, scale bar = 10 μm. **F** Quantitative analysis of the ratio of aggregated and monomeric JC-1 (*n* = 5). **G** Representative Mito-SOX fluorescence images indicate the level of mitochondrial oxidative stress, scale bar = 10 μm. **H** Quantitative analysis of mtROS production (*n* = 5). Data were presented as mean ± SD. One-way ANOVA was used for statistical analysis in (**B**), (**D**), (**F**), and (**H**).

It has been pointed out that Rock1 phosphorylates Drp1 to promote mitochondrial fission in response to myocardial ischemia/reperfusion injury [49]. Nevertheless, it remains cryptic whether Rnd3 reversed Dox-induced mitochondrial fission through Rock1. To delve deeper into the mechanistic role of Rock1 in pathological conditions, we employed siRNA-Rock1 to knockdown Rock1 expression in vitro. Western blot and PCR were performed to validate the knockdown efficiency (Supplementary Information 1: Fig. S11A) and the protein and mRNA levels of Rock1 were significantly downregulated (Supplementary Information 1: Fig. S11B and C), demonstrating the inhibition of Rock1 through present experimental manipulations. Under the condition of Rock1 downregulation, Western blot analysis revealed that the phosphorylation level of Drp1 at Ser616 was not further decreased in cardiomyocytes overexpressing Rnd3 (Fig. 6A, B). Despite the upregulation of Rnd3 alleviated Dox-elicited mitochondrial fragmentation as indicated by Mito-Tracker staining, this alleviation did not further present improvement after Rock1 deficiency (Fig. 6C, D). Similarly, the level of MMP and mtROS was not grossly altered by Rock1 knockdown in cardiomyocytes transfected with the Ad-Rnd3. (Fig. 6E–H).

In addition, we further examined whether Rock1 serves as the downstream regulator of Rnd3. Fasudil, a Rock1 inhibitor, was used to inhibit the expression of Rock1 in a variety of cardiovascular disease, such as diabetic cardiomyopathy [50] and myocardial ischemia/reperfusion injury [51, 52]. In the present study, Fasudil was employed in cardiomyocytes following Dox treatment to examine whether Rnd3 exerts its effects through Rock1 signaling. Subsequently, we used Western blot to verify the expression of Rock1 (Supplementary Information 1: Fig. S12A and B). Of note, Rnd3 overexpression did not further alleviate Dox induced mitochondrial disruption following the application of Fasudil (Supplementary Information 1: Fig. S12C–H). In summary, these results suggested that Rnd3 interacted with Rock1, and mitigated mitochondrial morphofunctional defects.

### Rock1 mediates the protective role of Rnd3 Against Dox-induced PANoptosis

To further elucidate the role of Rock1 in DIC, we performed dual staining with YP1 and PI in cardiomyocytes and examined the number of dead cells. We observed a remarkable decrease in the siRNA Rock group as compared to control group. Further, Rnd3 overexpression did not suppress Dox-triggered cell death in the condition of Rock1 inhibition, suggesting that Rnd3-induced protective effects on cardiomyocyte PANoptosis was reversed by Rock1 knockdown (Supplementary Information 1: Fig. S13A and B). Next, levels of PANoptosis-related proteins were evaluated using Western blot (Supplementary Information 1: Fig. S13C). The results indicated that Dox-induced PANoptosis was significantly inhibited by Rnd3 overexpression. However, such attenuation was effectively abrogated in the condition of Rock1 downregulation, indicating a crucial role for Rock1 in Rnd3-induced protective effects against Dox-induced PANoptosis (Supplementary Information 1: Fig. S13D–M). These findings provided insights into the molecular mechanisms underlying the protective effects of Rnd3 against Dox-induced cardiomyocyte PANoptosis and highlighted the importance of Rock1 in this process.

### Rock1 was involved in Rnd3-mediated Dox-induced cardiac remodeling and dysfunction

**Fig. 7:**
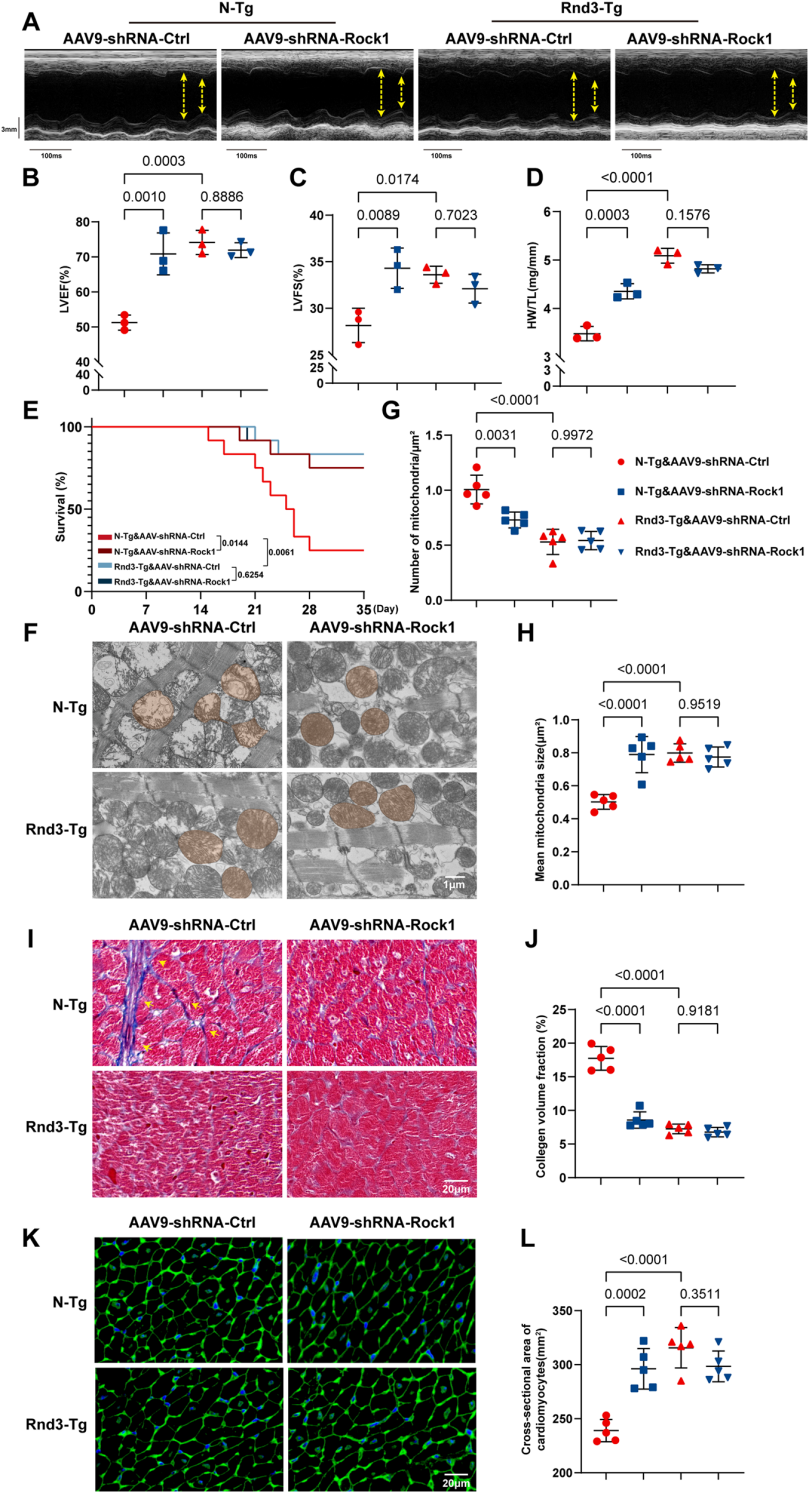
Rock1 knockdown abolished the effects of Rnd3 on Dox-induced cardiac remodeling and dysfunction. **A** Representative M-mode recordings of echocardiography of N-Tg or Rnd3-Tg mice injected with AAV9-shRNA-Control or AAV9-shRNA-Rock1, and then treated with Dox. **B**, **C** Quantitative analysis of LVEF and LVFS of heart (*n* = 3). **D** Quantitative analysis of HW/TL (*n* = 3). **E** Survival curves of the mice during the 4 W period (*n* = 12). **F** Representative ultrastructural images of Mitochondria obtained by transmission electron microscope following treatment with Dox, scale bar = 1 μm. **G**, **H** Quantitative analysis of mitochondrial number per field and mean mitochondrial size (*n* = 5). **I**, **J** Representative images and quantitative analysis of masson trichrome staining in the peripheral of heart following Dox treatment, scale bar = 20 μm. **K**, **L** Representative WGA staining images and quantification of indicated mice (*n* = 5). Data were presented as mean ± SD. One-way ANOVA was used for statistical analysis in (**B**)–(**G**), (**H**), (**J**), and (**L**).

We next explored whether Rock1 was required for the cardioprotective effect of Rnd3 against cardiac structural remodeling, an adeno-associated virus 9 (AAV9) was employed to downregulate Rock1 expression in vivo. We first randomly delivered AAV9-shRNA-Rock1 or AAV9-shRNA-Control into N-Tg and Rnd3-Tg mice by intramyocardial injection, and employed immunofluorescence staining to examine the transfection efficiency of adeno-associated virus. The results revealed that adeno-associated virus were effectively introduced into cardiomyocytes, achieving a notable transfection efficiency (Supplementary Information 1: Fig. S14A). The effectiveness of Rock1 knockdown in myocardial tissues was subsequently verified using Western blot, ensuring the successful downregulation of Rock1 in vivo (Supplementary Information 1: Fig. S14B and C). Subsequently, echocardiography analysis indicated that Rnd3-Tg enhanced parameters such as LVEF, and LVFS, following the Dox challenge. Notably, Rnd3 overexpression did not further reduce cardiac dysfunction after Rock1 knockdown (Fig. 7A–C). A similar phenomenon is seen in HW/TL and survival rate (*P* < 0.05), denoting that Rock1 was involved in the protection of Rnd3 in vivo (Fig. 7D, E). Transmission electron microscopy revealed that Rnd3 supply did not enhance mitochondrial protection following Rock1 downregulation, as indicated by the number of mitochondrial fragmentation (Fig. 7F–H). In cardiac morphology, masson trichrome staining revealed that the fibrosis area, which was overtly decreased by Rnd3 upregulation, did not show any notable differences when Rock1 was suppressed in Dox-treated mice (Fig. 7I, J). Likewise, WGA staining showed an increased cross-sectional area of cardiomyocytes in Rnd3-Tg mice under Dox treatment. However, Rnd3 overexpression did not pronouncedly enhance the restoration of the cardiomyocyte size following AAV9-shRNA-Rock1 delivery (Fig. 7K, L).

To further explore the role of Rock1 in Rnd3 mediated effects against DIC, Fasudil was used to inhibit Rock1 expression in vivo by intraperitoneal injection. The effectiveness of Rock1 knockdown in myocardial tissues was subsequently verified using Western blot (Supplementary Information 1: Fig. S15A and B). Of note, Fasudil application mitigated cardiac dysfunction, alleviated cardiac fibrosis, while enlarged cross-sectional area of cardiomyocytes in the condition of Dox treatment. Consistent with the above results, Rnd3 overexpression did not further alleviate cardiac remodeling following Fasudil injection as indicated by echocardiography, masson trichrome staining and WGA staining (Supplementary Information 1: Fig. S15C–I). Based on these findings, it appeared that Rock1 plays a crucial role in the protective effects of Rnd3 in DIC.

## Discussion

Despite its widespread use as an effective chemotherapeutic agent against various cancers, Dox has a significant limitation due to its cardiotoxicity [3, 53]. Recent studies have unveiled that rodents exhibiting Rnd3 haploinsufficiency often manifested a pathological state characterized by pressure overload [54]. Importantly, emerging evidence has demonstrated that Rnd3 overexpression effectively improves myocardial structure and function, providing a promising therapeutic avenue for the management of cardiovascular diseases [55].

In this study, gene expression profiles of left ventricle tissues from Dox or saline-treated mice indicated that Rnd3 may serve as a potential key regulator of DIC. A significant downregulation of Rnd3 in response to Dox insult was noted using Western blot, PCR, immunofluorescence, and immunohistochemistry. Cardiomyocyte-specific Rnd3 overexpression mitigated Dox-induced cardiac dysfunction, as evidenced by increased LVEF and LVFS. Cardiomyocyte-specific Rnd3 overexpression also enhanced HW/TL and inhibited cardiac fibrosis, atrophy, and cardiac enzyme release in response to Dox challenge. Importantly, Rnd3 overexpression improved the survival rate and restricted mean survival time of Dox-exposed mice, suggesting that cardiac Rnd3 overexpression ameliorated Dox-induced cardiac dysfunction and remodeling in mice.

Previous studies have established that Dox disrupts mitochondrial dynamics, particularly by skewing the balance between mitochondrial fission and fusion, leading to enhanced susceptibility to cell death [56–58]. Accumulating evidence also indicated that mitochondrial integrity is crucial for cellular survival and highlights the therapeutic potential of targeting mitochondrial fission to mitigate the adverse effects of Dox in cardiomyocytes [58–60]. The current study revealed profound mitochondrial cristae damage following Dox challenge as evidenced by the transmission electron micrograph. Interestingly, Rnd3 overexpression inhibited Dox-induced mitochondrial fission, upregulated MMP, while reduced mtROS production.

The pivotal role of mitochondrial fission and its regulation by Drp1 phosphorylation is well established in the context of cellular responses to stress [28, 61, 62]. Given that Drp1 is a well-established regulator of mitochondrial fission, we proceeded to investigate whether Rnd3-regulated mitochondrial fission is dependent on Drp1 phosphorylation. The results indicated that Rnd3 overexpression markedly attenuated Drp1 phosphorylation specifically at Ser616. To further verify this conclusion, we specifically overexpressed a Drp1 phosphomimetic (Drp1 S616D) in cardiomyocytes by delivering AAV9-Drp1 S616D into N-Tg mice or Rnd3-Tg mice. Echocardiography analysis and transmission electron microscopy indicated that AAV9-Drp1 S616D injection offsets Rnd3-offered protection against cardiac dysfunction and mitochondrial injury following Dox treatment. These findings provide solid evidence that Rnd3 serves as a protective factor against Dox-induced mitochondrial disruption by inhibiting Drp1 phosphorylation at Ser616. The cardioprotective role of Rnd3 in DIC significantly contributes to the current understanding of mitochondrial dysfunction as a central mechanism of action in Dox-evoked cardiac damage.

The concept of PANoptosis, which encompasses apoptosis, necroptosis, and pyroptosis [63, 64], provides a comprehensive framework to understand the multifaceted nature of DIC. Ample evidence also indicates that deranged mitochondrial dynamics may trigger programmed cell death, encompassing apoptosis, necroptosis, and pyroptosis [65–67]. To gain a deeper understanding of how Rnd3 ameliorates cardiomyocyte fate, RNA-sequencing analysis was performed in cardiomyocytes transfected with Ad-Rnd3 or Ad-Control following Dox treatment. GSEA analysis revealed significant differences in gene expression associated with apoptosis, necroptosis, and pyroptosis pathways between the Ad-Rnd3 group and the Ad-Control group. These findings suggest that Rnd3 may exert its protective effects by modulating PANoptosis.

The PANoptosome acts as a complex assembly of multiple proteins, including sensors such as AIM2, ZBP1, and NLRP3, which orchestrate the simultaneous occurrence of pyroptosis, apoptosis, and necroptosis [10, 36, 37]. The present study revealed that Dox significantly enhanced PANoptosis in cardiomyocytes, as indicated by elevated levels of PANoptosome components such as AIM2, ZBP1, and NLRP3. Conversely, Rnd3 overexpression inhibited the levels of activated markers associated with PANoptosis, suggesting its potential protective role in mitigating Dox-induced cell death in the heart. The reduction of PANoptosis in the presence of Rnd3 overexpression highlights its pivotal role in mitigating Dox-induced mitochondrial fragmentation and dysfunction. The present results suggest that by stabilizing mitochondrial fission, Rnd3 serves as a crucial modulator that intercepts the cascade prompting PANoptosis, thereby offering a potential therapeutic target to reduce DIC.

Li and colleagues indicated that Rock1 is a kinase regulating Drp1 activity at Ser616 [49]. Earlier work also unveiled the capacity of Rock1 to control Drp1 phosphorylation in the context of neoplastic conditions [68]. In the present study, co-localization and molecular docking analysis revealed a direct interaction between Rnd3 and Rock1, which may impede Drp1 phosphorylation at Ser616, thus alleviating mitochondrial fission and dysfunction. Furthermore, co-immunoprecipitation assay revealed a tight combination of Rnd3 and Rock1 which located in the cytoplasm of cardiomyocyte. To further elucidate the involvement of Rock1 in Rnd3-offered protection against DIC, siRNA-Rock1 vector and Fasudil were constructed to inhibit Rock1 expression and its activity. As expected, Rock1 downregulation induced a decline in the phosphorylation level of Drp1 at Ser616. Rnd3 replenishment did not modify the effects of Rock1 on Drp1 phosphorylation, mitochondrial fission, and mitochondrial dysfunction elicited by the Dox challenge. Interestingly, we observe the same pattern of results in mtROS production and MMP in cardiomyocytes in the face of Dox exposure. These findings suggests that Rnd3 directly combines with Rock1 in cytoplasm, and inhibiting Drp1 phosphorylation at Ser616, thereby attenuating mitochondrial dysfunction. By using an adeno-associated virus 9 (AAV9) which downregulates Rock1 expression in vivo, we also demonstrated that Rock1 knockdown offset Rnd3 offered protective effects against cardiac dysfunction and mitochondrial injury. Consistently, Rock1 inhibition by Fasudil also revealed that Rock1 serves as a downstream regulator of Rnd3. These results indicated that Rnd3/Rock1 signaling mediates Drp1 phosphorylation in cardiomyocytes thus halting the progression of DIC.

## Conclusions

This study demonstrated a crucial role for Rnd3 in attenuating DIC by inhibiting PANoptosis in a Rock1/Drp1/mitochondrial fission-dependent manner. Specifically, Rnd3 binds to Rock1 in cytoplasm, thereby reducing Drp1 phosphorylation and preventing excessive mitochondrial fission, ultimately alleviating Dox-induced PANoptosis. These findings suggest that Rnd3 could serve as a viable strategy to mitigate DIC, potentially improving treatment outcomes for cancer patients.

## Supplementary information

Supplementary Information 1

Supplementary Information 2

Supplementary Information 3

Supplementary Information 4

The original Western blot bands

## Data Availability

All data generated or analyzed during this study are included in this published article and its Supplementary Information or from the corresponding author upon reasonable request.
