# Supplementary material for: Rnd3 protects against doxorubicin-induced cardiotoxicity through inhibition of PANoptosis in a Rock1/Drp1/mitochondrial fission-dependent manner
Source: Cell Death Dis. 2025 Jan 4;16(1):2. doi: 10.1038/s41419-024-07322-0 (PMC11700182; doi:10.1038/s41419-024-07322-0)
Supplement: Supplementary file 1 — Supplementary Information 1 [file 41419_2024_7322_MOESM1_ESM.docx]

**Rnd3 Protects against Doxorubicin-Induced Cardiotoxicity through Inhibition of PANoptosis in a Rock1/Drp1/Mitochondrial Fission-Dependent Manner**

Wen Ge^1, 2^, Xiaohua Zhang^1, 2^, Jie Lin^1, 2^, Yangyang Wang^1^, Xiao Zhang^1^, Yu Duan^1^, Xinchun Dai^1^, Jiye Zhang^1^, Yan Zhang^1^, Mengyuan Jiang^1^, Huanhuan Qiang^1^, Zhijing Zhao^1, #^, Xuebin Zhang^1,#^, Dongdong Sun^1, #^

^1^ Department of Cardiology, Xijing Hospital, Fourth Military Medical University, Xi’an, Shaanxi, China

^2^ These authors contributed equally: Wen Ge, Xiaohua Zhang, Jie Lin.

^#^ Corresponding authors: 127 West Changle Road, Department of Cardiology, Xijing Hospital, Fourth Military Medical University, Xi'an, Shaanxi 710032, China

E-mail: zhao_zhj@126.com; Zhangxuebin_wy@163.com; wintersun3@fmmu.edu.cn.

**1. Tables (Table S1–S5)**

**2. Figures (Figure S1–S15)**

**Table 1 Primer sequences for genotyping**

| **Mouse** | **Forward primers (5’-3’)** | **Reverse primers (5’-3’)** |
| --- | --- | --- |
| Rnd3 (5 arm LoxP) | TCCAGGTAGATAGGGGGATAGATG | TGGGGAGAGTGAAGCAGAACG |
| Rnd3 (3 arm LoxP) | GCCACTCCCACTGTCCTTTCC | CCTTACCACACACTACCCTTCCCT |
| Myh6-Cre | TCTATTGCACACAGCAATCCA | CCAGCATTGTGAGAACAAGG |

**Table 2 Primary antibodies used for Western blot, immunoprecipitation, immunofluorescence, and immunohistochemistry**

| **Antibody** | **Supplier** | **Catalog No.** | **Working dilutions** |
| --- | --- | --- | --- |
| Rnd3 | Santa Cruz | sc-53874 | WB: 1/1000 |
| Rnd3 | Affinity | #DF12311 | IF: 1/200  IHC:1/200  IP:1/50 |
| Drp1 | Abcam | AB184247 | WB: 1/2000  IP:1/30 |
| Drp1 (phospho S616) | Abcam | AB314755 | WB: 1/1000 |
| Rock1 | Abcam | AB134181 | WB:1/1000  IP:1/100 |
| Rock1 | Proteintech | 21850-1-AP | IF: 1/200 |
| RhoA | Abcam | AB187027 | WB:1/1000 |
| AIM2 | Cell Signaling Technology | #63660 | WB: 1/1000 |
| ZBP1 | Proteintech | 13798-1-AP | WB: 1/1000 |
| NLRP3 | Cell Signaling Technology | #15101 | WB: 1/1000 |
| Cleaved caspase 3 | ABclone | A11021 | WB: 1/1000 |
| Caspase-3 | ABclone | A2156 | WB: 1/1000 |
| Cleaved caspase-8 | Cell Signaling Technology | #9496 | WB: 1/1000 |
| Caspase-8 | ABclonal | A0215 | WB: 1/1000 |
| Cleaved caspase-1 | ABclonal | A11021 | WB: 1/1000 |
| Caspase-1 | ABclonal | A18646 | WB: 1/1000 |
| Cleaved GSDMD | ABclonal | A24059 | WB: 1:3600 |
| GSDMD | ABclonal | A20728 | WB: 1/1000 |
| p-MLKL | ABclonal | AP1173 | WB: 1/1000 |
| t-MLKL | ABclonal | AP1244 | WB: 1/1000 |
| P-RIPK1 | ABclonal | AP1230 | WB: 1/1000 |
| t-RIPK1 | ABclonal | A7414 | WB: 1/1000 |
| p-RIPK3 | ABclone | AP1408 | WB: 1/1000 |
| t-RIPK3 | ABclone | A5431 | WB: 1/1000 |
| β-Tubulin | Proteintech | 10094-1-AP | WB: 1/1000 |

WB: Western blot, IF: Immunofluorescence, IP: Immunoprecipitation

**Table 3 Primer sequences for RT-PCR**

| **Mouse** | **Forward 5’ to 3’** | **Reverse 5’ to 3’** |
| --- | --- | --- |
| Rnd3 | TTTCGCACATGCCTAGCAGA | CAGATATTCCCGCGTCCTCC |
| Rock1 | AACATGCTGCTGGATAAATCTGG | TGTATCACATCGTACCATGAAGCT |
| GAPDH | GGTGAAGGTCGGTGTGAACG | CTCGCTCCTGGAAGATGGTG |

**Table 4 Protein-protein docking** of the top 10 models using HDOCK

| **Rank** | 1 | 2 | 3 | 4 | 5 | 6 | 7 | 8 | 9 | 10 |
| --- | --- | --- | --- | --- | --- | --- | --- | --- | --- | --- |
| **Docking Score** | -237.94 | -226.32 | -215.92 | -215.46 | -214.91 | -211.31 | -209.66 | -204.74 | -203.48 | -201.31 |
| **Confidence Score** | 0.8531 | 0.8215 | 0.7889 | 0.7874 | 0.7855 | 0.7732 | 0.7673 | 0.7493 | 0.7445 | 0.7362 |

**Table 5 Protein-protein docking** of the top 10 models using Cluspro

| **Cluster** | **Members** | **Representative** | **Weighted Score** |
| --- | --- | --- | --- |
| **0** | 81 | Center | -1059.5 |
|  |  | Lowest Energy | -1180.2 |
| **1** | 72 | Center | -1046.1 |
|  |  | Lowest Energy | -1296.6 |
| **2** | 42 | Center | -969 |
|  |  | Lowest Energy | -1031.1 |
| **3** | 39 | Center | -1116.6 |
|  |  | Lowest Energy | -1116.6 |
| **4** | 37 | Center | -986.1 |
|  |  | Lowest Energy | -1065.8 |
| **5** | 34 | Center | -987.3 |
|  |  | Lowest Energy | -1036.9 |
| **6** | 30 | Center | -1015.5 |
|  |  | Lowest Energy | -1015.5 |
| **7** | 28 | Center | -908.6 |
|  |  | Lowest Energy | -1014.3 |
| **8** | 27 | Center | -1031.2 |
|  |  | Lowest Energy | -1031.2 |
| **9** | 26 | Center | -901.3 |
|  |  | Lowest Energy | -1049.3 |
| **10** | 25 | Center | -1033.8 |
|  |  | Lowest Energy | -1066.8 |

**2. Figures**

**
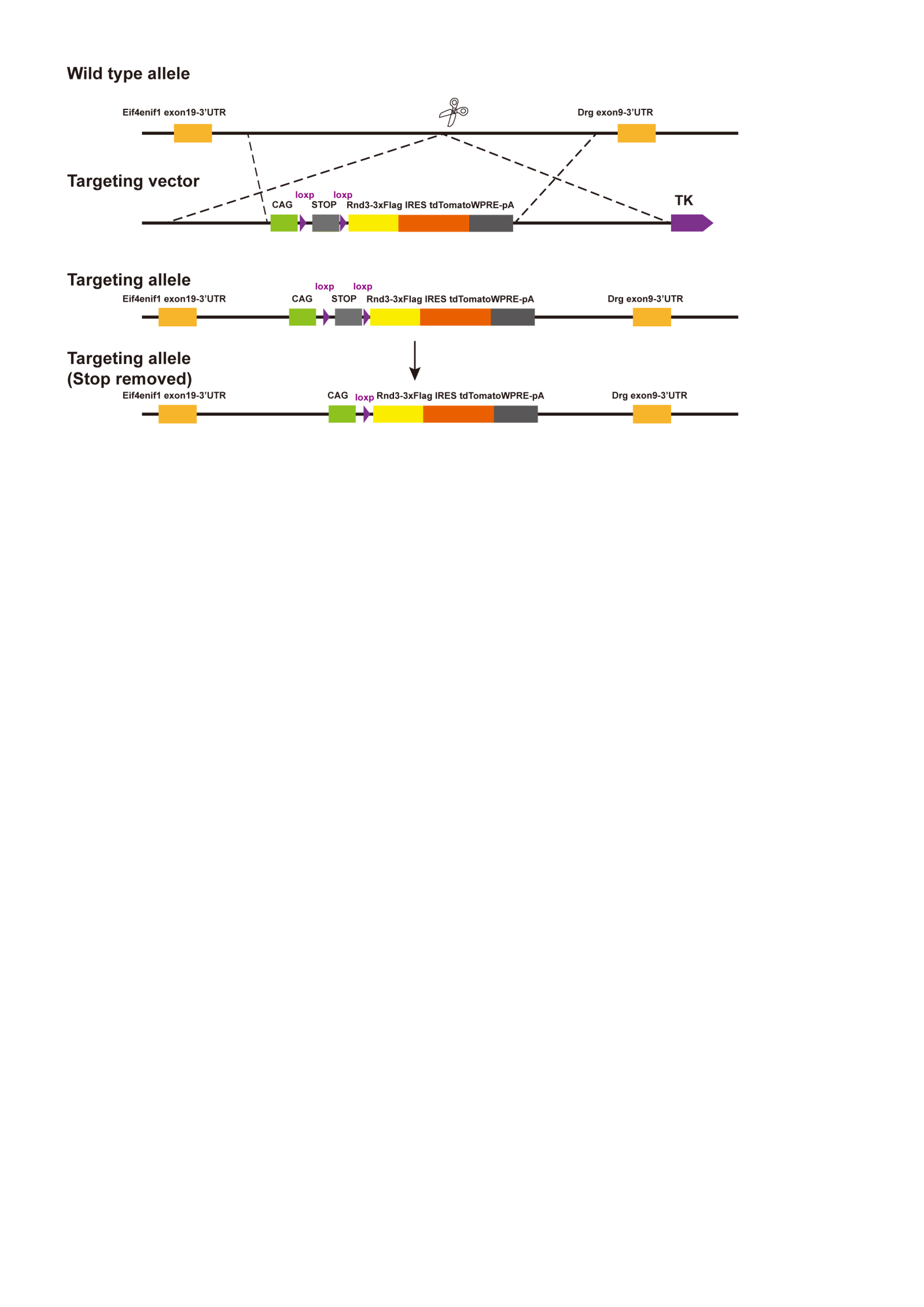
Fig. S1. Schematic diagram of transgenic mice**

Gene: Rnd3 (Ensembl ID: ENSMUSG00000017144)

Gene: Hipp11 locus (Located at the junction of genes Eif4enif1 (Ensembl ID: ENSMUSG00000020454) and Drg1 (Ensembl ID: ENSMUSG00000020457)

Site: [Chromosome 11: 3,245,2](http://asia.ensembl.org/Mus_musculus/Location/View?db=core;g=ENSMUSG00000086429;r=6:113067428-113077333)59


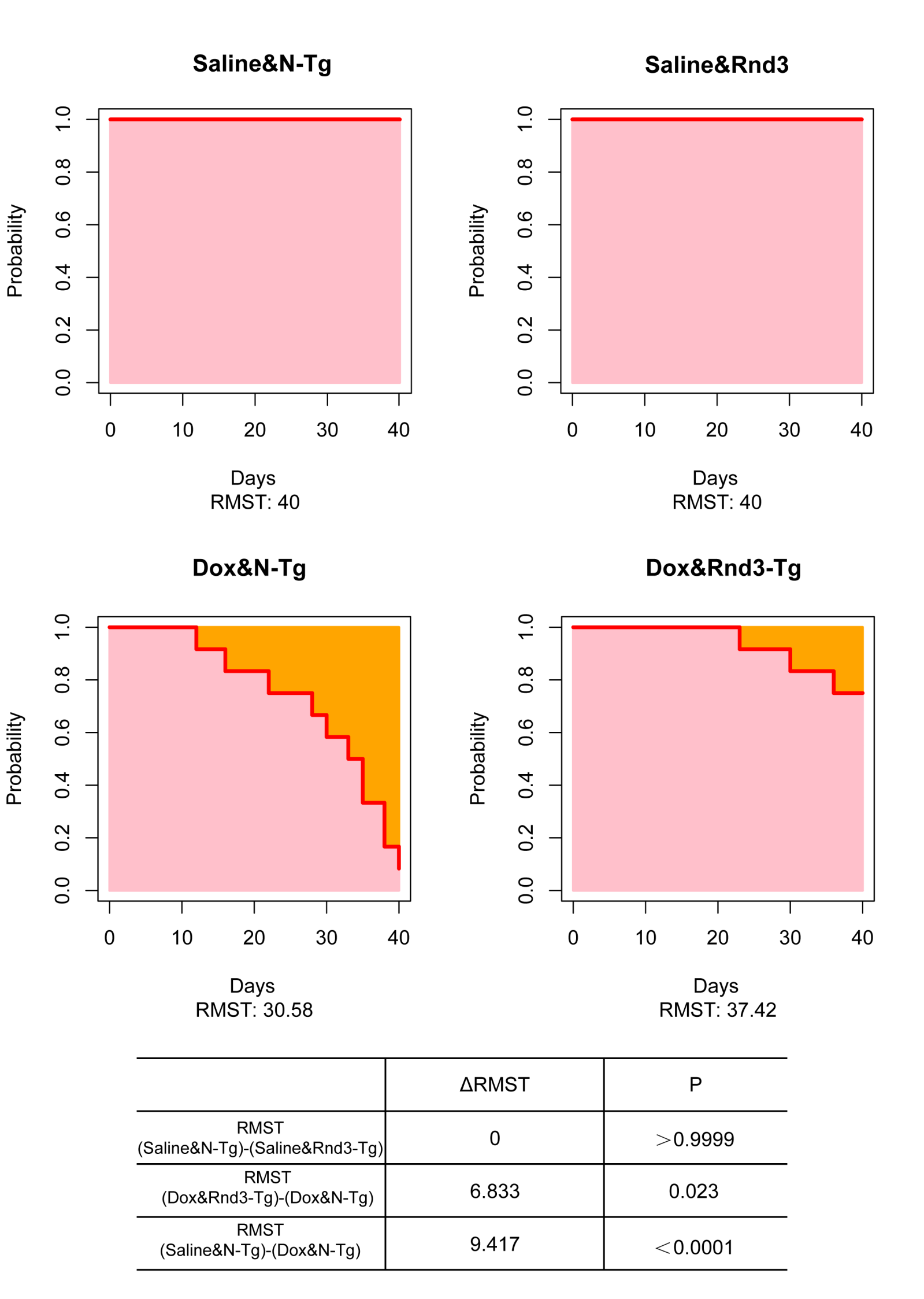


**Fig. S2.** **Rnd3 overtly improved the restricted survival rate in DIC.** Compared with the N-Tg mice, restricted mean survival time (RMS time) was significantly increased in Rnd3-Tg mice following Dox treatment from 30.58 days to 37.42 days.

**
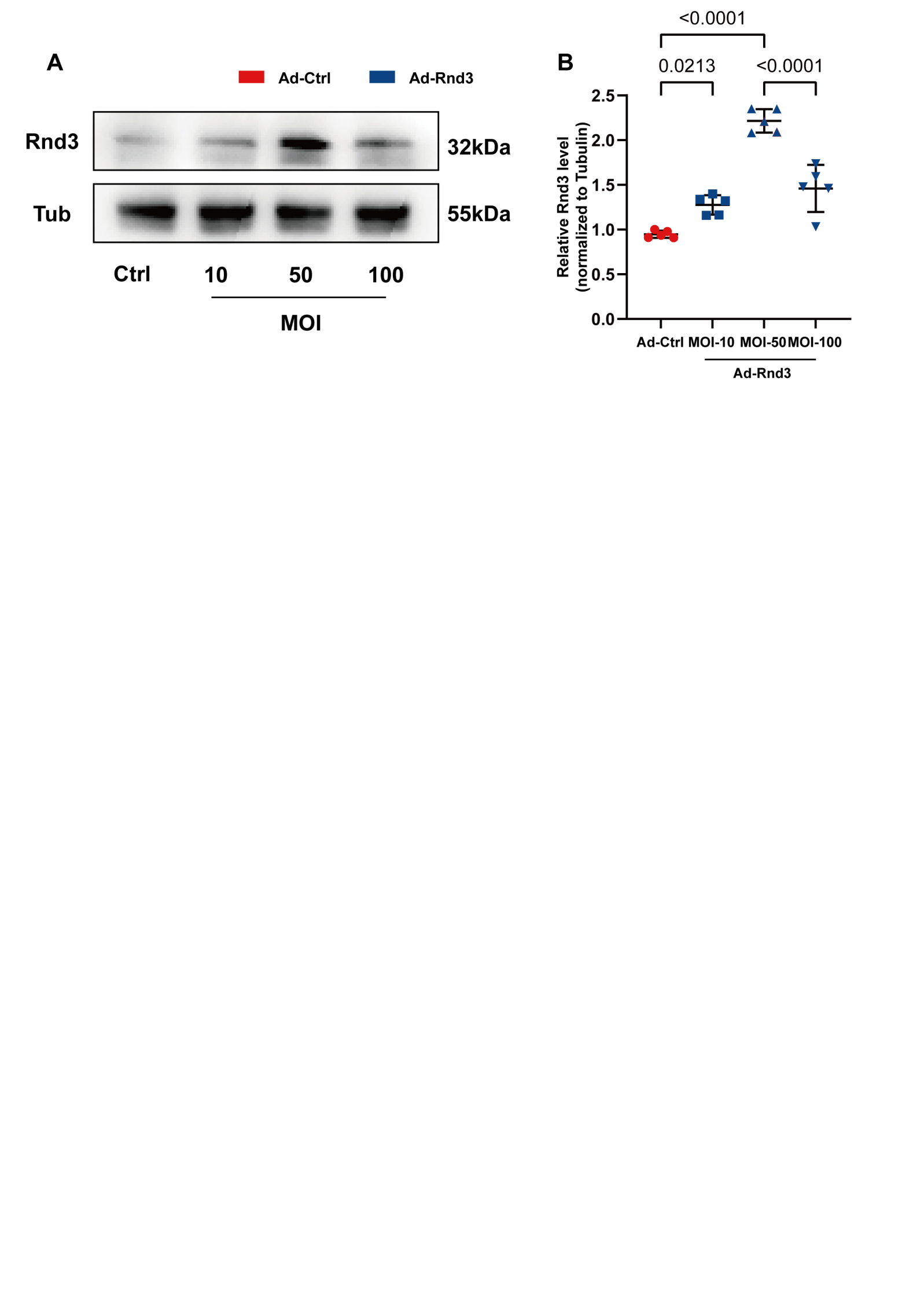
**

**Fig. S3. The transfection efficiency was evaluated across different MOIs, with the highest Rnd3 expression observed at 50 MOI 48 hours post-transfection with Ad-Rnd3.**

**A** Representative Western blot images of Rnd3 protein levels in cardiomyocytes. **B** Quantitative analysis of Rnd3 protein expression (n = 5). Data were presented as mean ± SD. One-way ANOVA was used for statistical analysis in **B**.


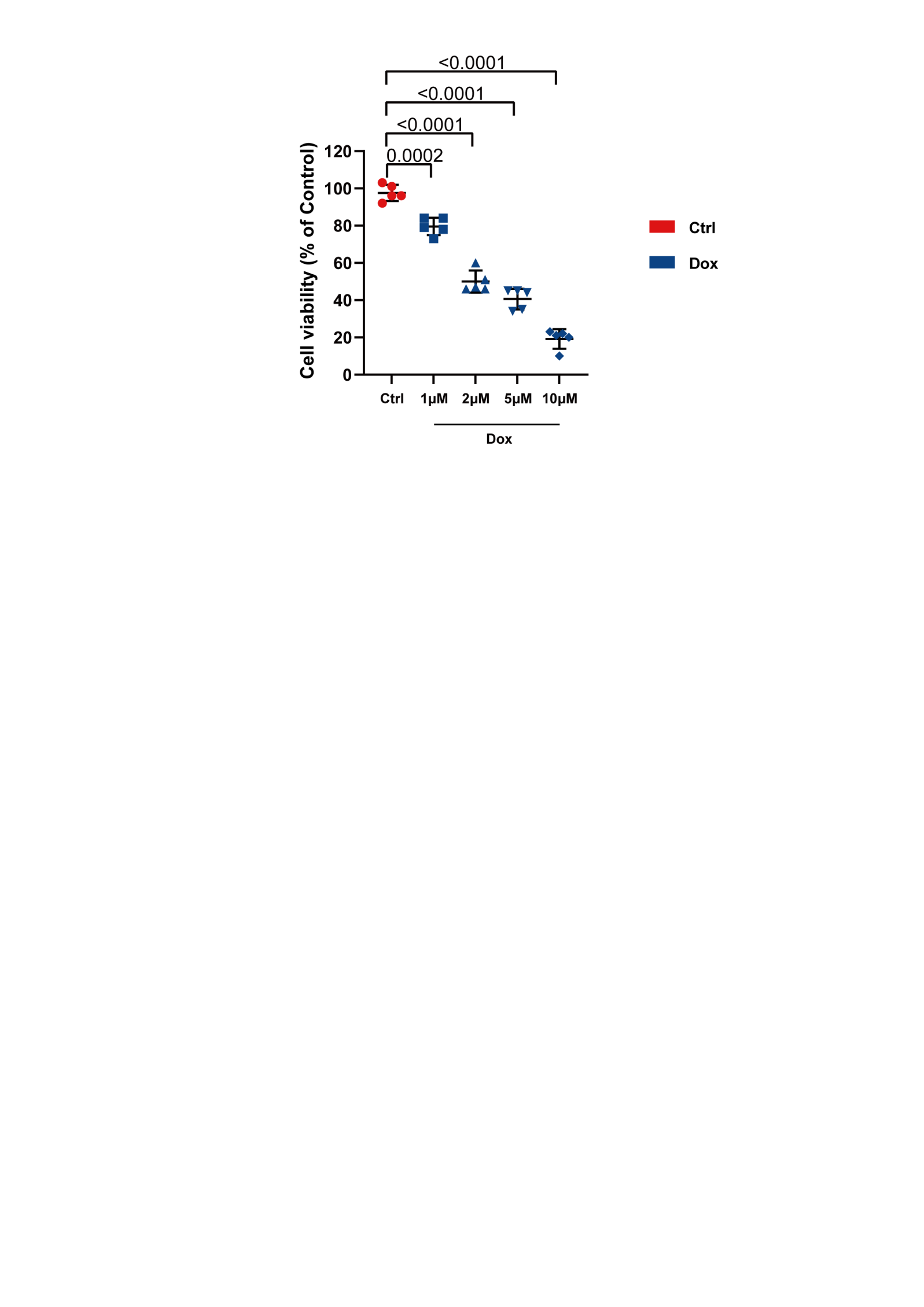


**Fig. S4. The vitality of cardiomyocytes under different concentrations of Dox.** The cell viability of cardiomyocytes was significantly decreased after 24 hours following Dox challenge. CCK-8 assay showed that cell viability reduced about 50% in the dose of 2μM (n = 5). Data were presented as mean ± SD. One-way ANOVA was used for statistical analysis.

**
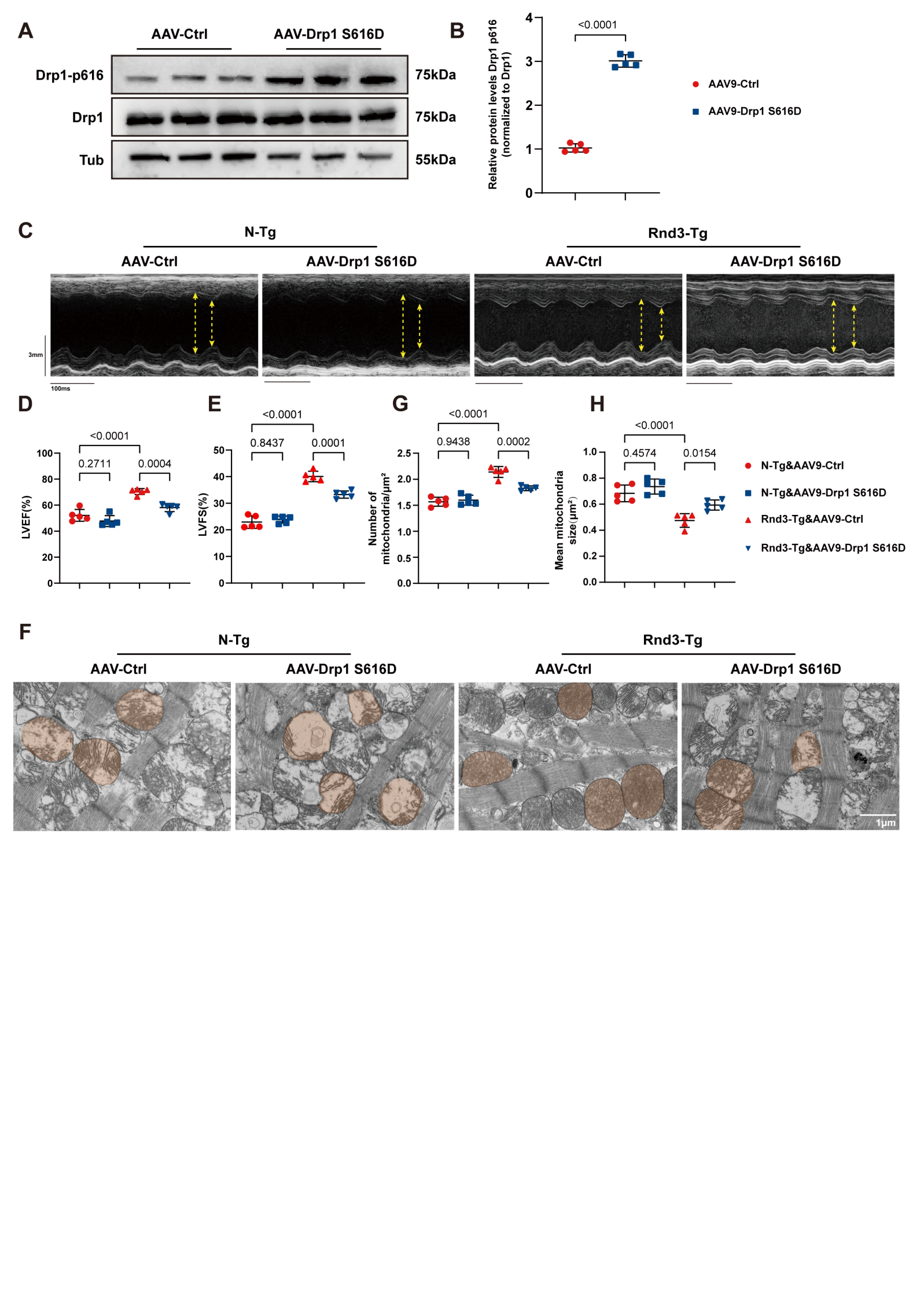
**

**Fig. S5. Drp1 S616 overexpression aggarvates Dox-induced cardiac dysfunction and mitochondrial dysfunction**

**A** Representative Western blot images of the efficiency of Drp1 S616D overexpression in N-Tg or Rnd3-Tg mice following Dox treatment. **B** Quantitative analysis of Drp1-p616/Drp1 protein expression (n = 5). **C** Representative M-mode echocardiographic imaging of heart in N-Tg or Rnd3-Tg mice following Dox treatment. **D-E** Analysis of LVEF and LVFS of heart (n = 5). **F** Representative images of Mitochondria obtained by transmission electron microscope following treatment with Dox. **G-H** Quantitative analysis of mitochondrial number per field and mean mitochondrial size (n = 5). Data were presented as mean ± SD. Student’s t-test were used for statistical analysis in **B**. One-way ANOVA was used for statistical analysis in **D**, **E**, **G** and **H**.

**
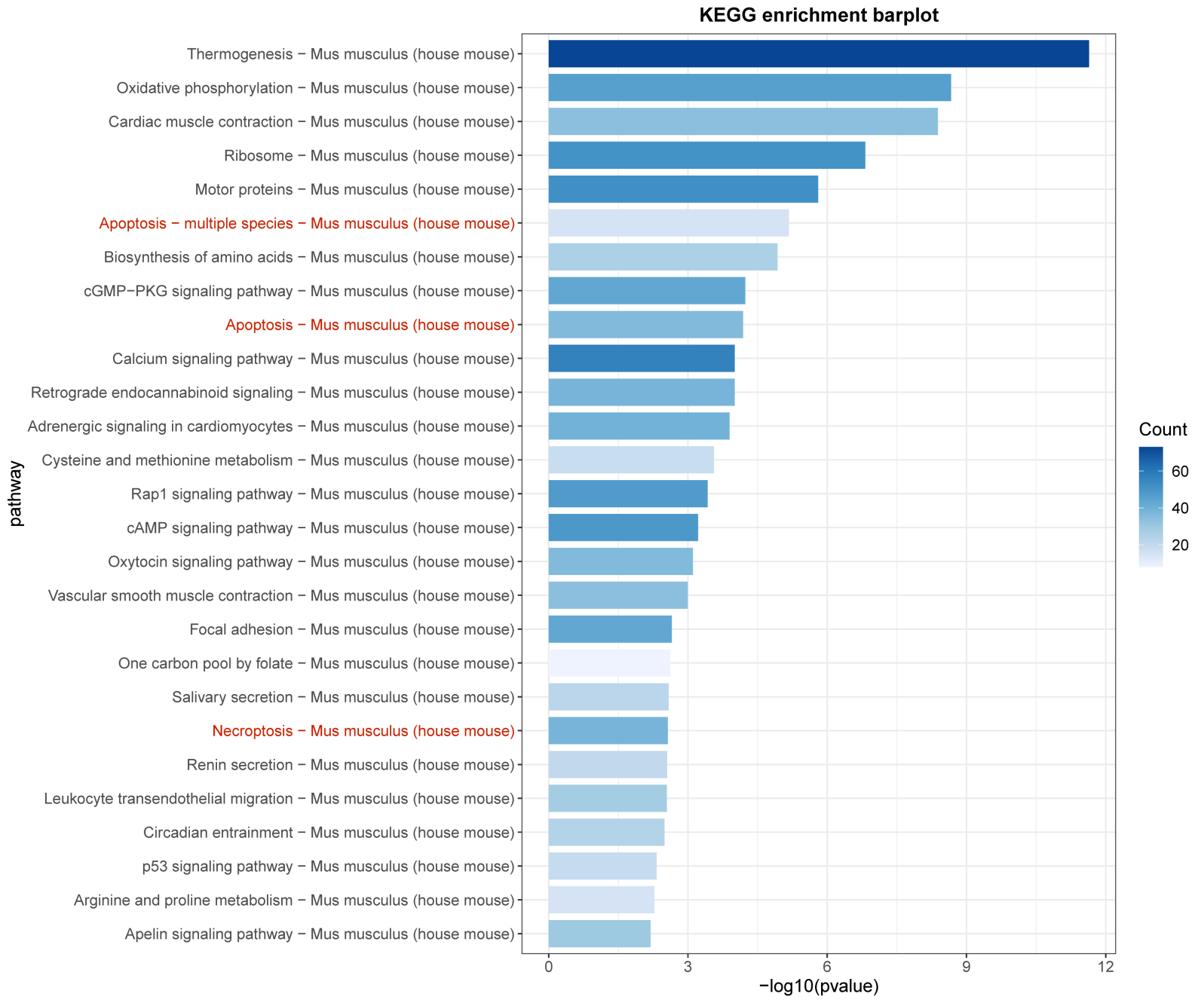
**

**Fig. S6. Kyoto Encyclopedia of Genes and Genomes (KEGG) enrichment analysis.** KEGG pathway analysis revealed that Rnd3 overexpression in cardiomyocytes were significantly enriched in multiple cell death pathways.


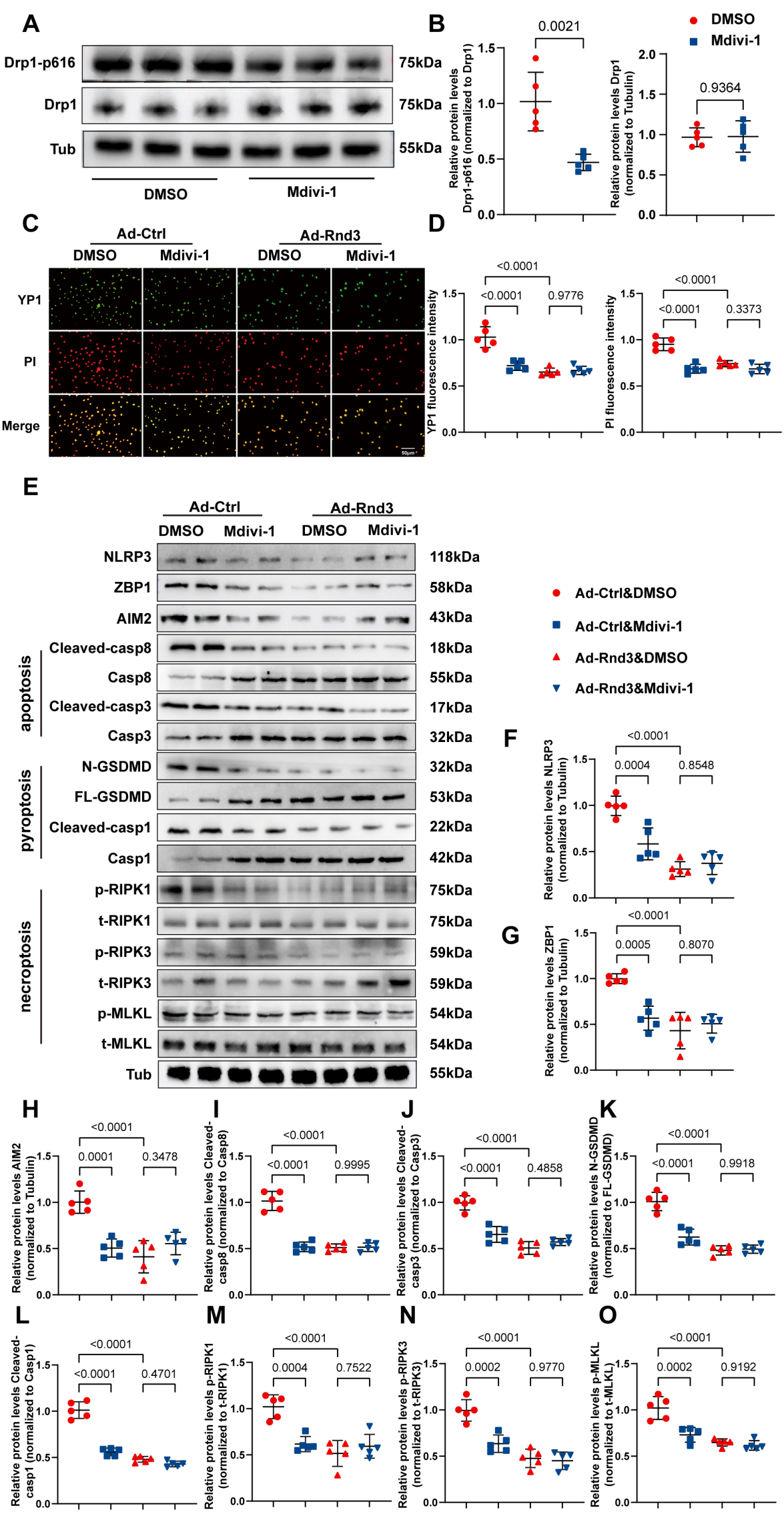


**Fig. S7. Inhibition of mitochondrial fission mitigates Dox-induced cardiomyocyte PANoptosis.**

**A** Representative Western blot images of Drp1 and Drp1-p616 protein levels in cardiomyocytes treated with DMSO or Mdivi-1 for 24 h and then treated with Dox for 24 h. **B** Quantitative analysis of Drp1 and Drp1-p616/Drp1 protein expression (n = 5). **C** Representative immunofluorescence images showing YP1-positive cells (green) which may undergo apoptosis or necroptosis and PI-positive cells (red) which may undergo apoptosis, necroptosis, or pyroptosis. Cardiomyocytes were infected with Ad-Control or Ad-Rnd3, and then treated with DMSO or Mdivi-1 respectively for 24h followed by 24h exposure to 2µM Dox, scale bar = 50μm. **D** Quantitative analysis of YP1 and PI-positive cells (n = 5). **E** PANoptosis-related proteins were assessed by Western blot. Cardiomyocytes infected with Ad-Control or Ad-Rnd3, and then treated with DMSO or Mdivi-1 for 24h followed by 24h exposure to 2µM Dox. **F-O** Quantitative analysis of PANoptosis-related proteins expression (n = 5). Data were presented as mean ± SD. Student’s t-test were used for statistical analysis in **B**. one-way ANOVA was used for statistical analysis in **D**, **F**, **G**, **H**, **I**, **J**, **K**, **L**, **M**, **N** and **O**.

**
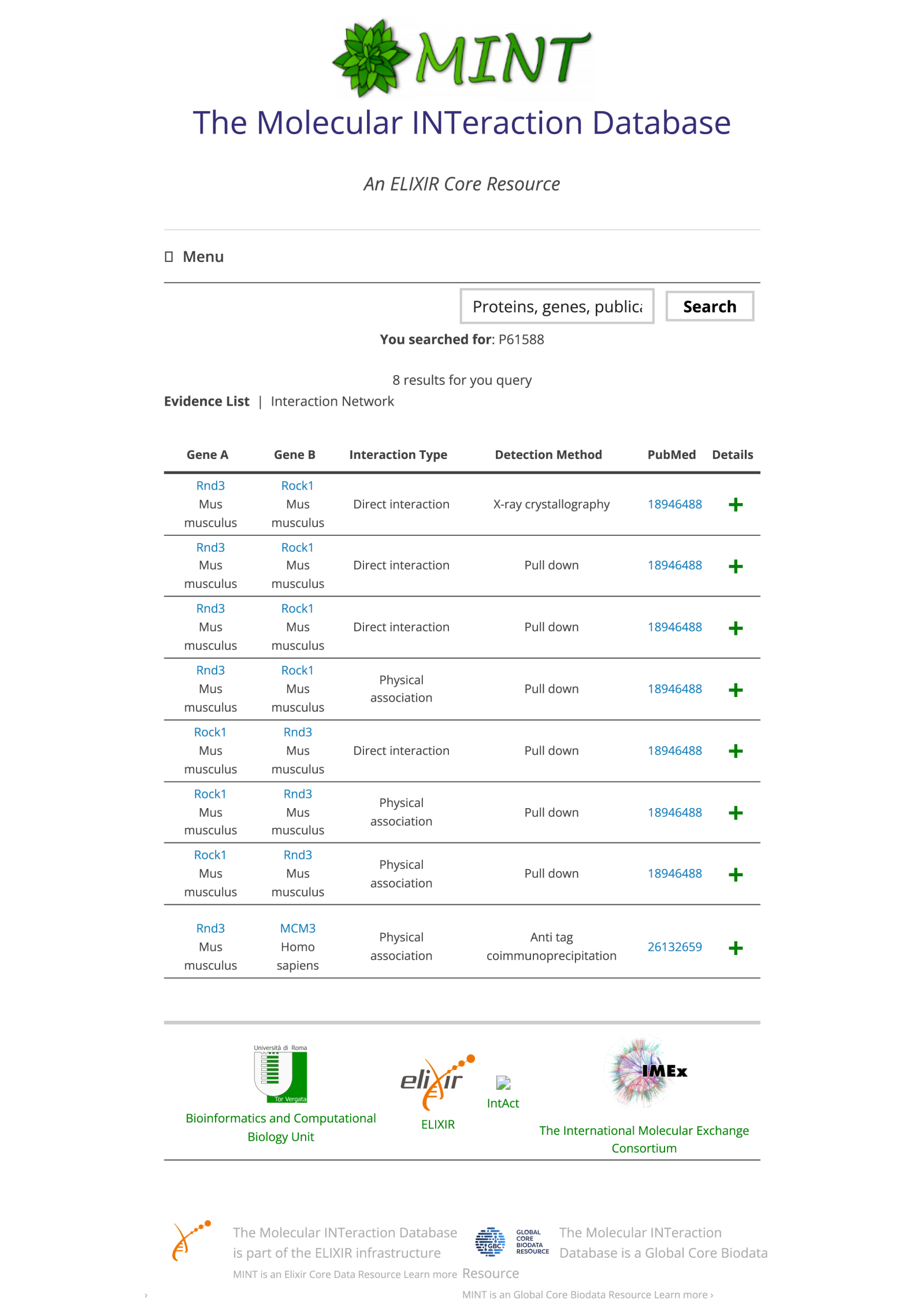
**

**Fig. S8. Protein-protein interaction shows Rock1 as a potential interacting protein of Rnd3 according to MINT databases.**

**
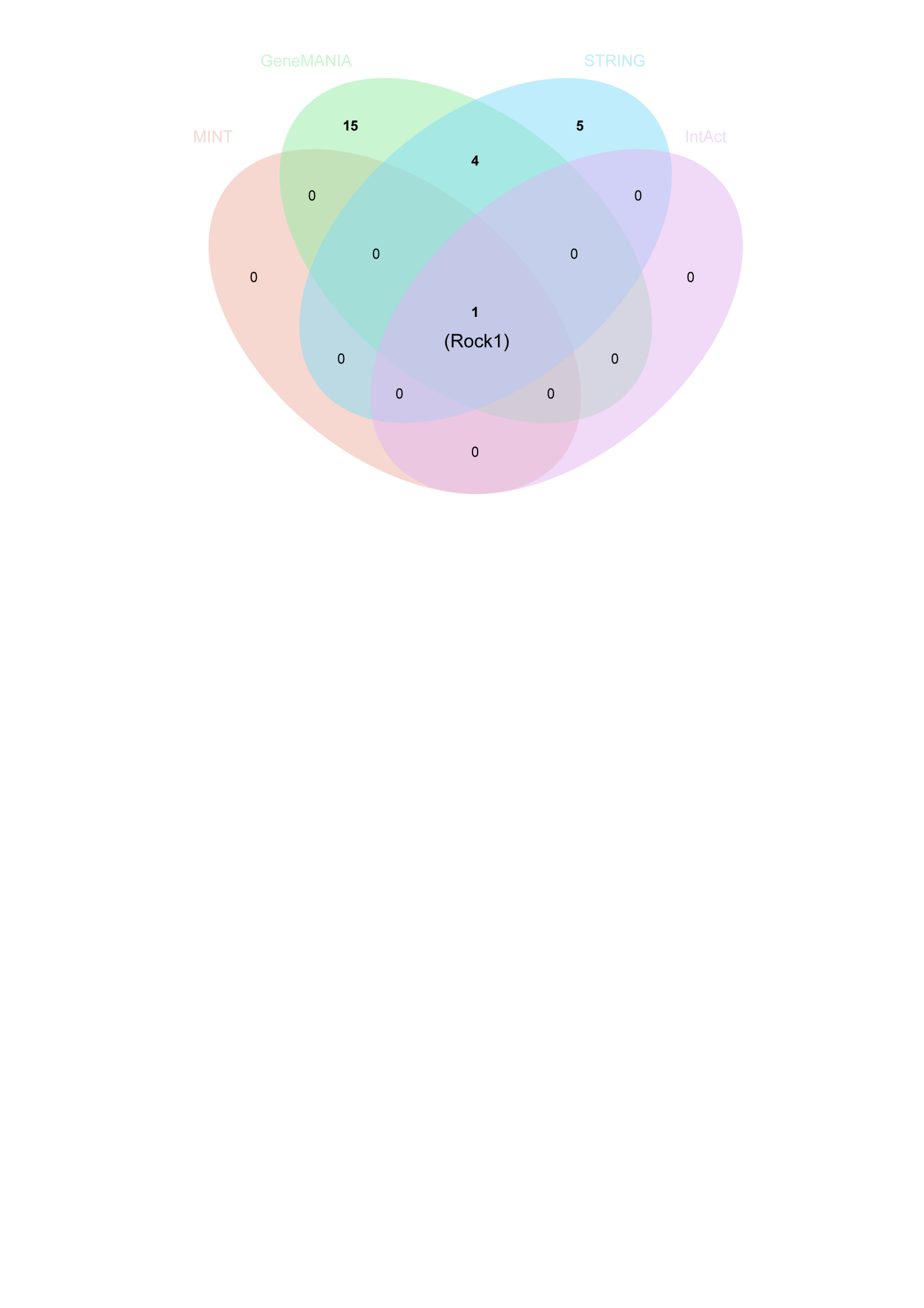
**

**Fig. S9. Venn diagrams showed that Rock1 was a potential interacting protein of Rnd3 according to Protein-protein interaction (IntAct, STRING, GeneMANIA, and MINT)**

**
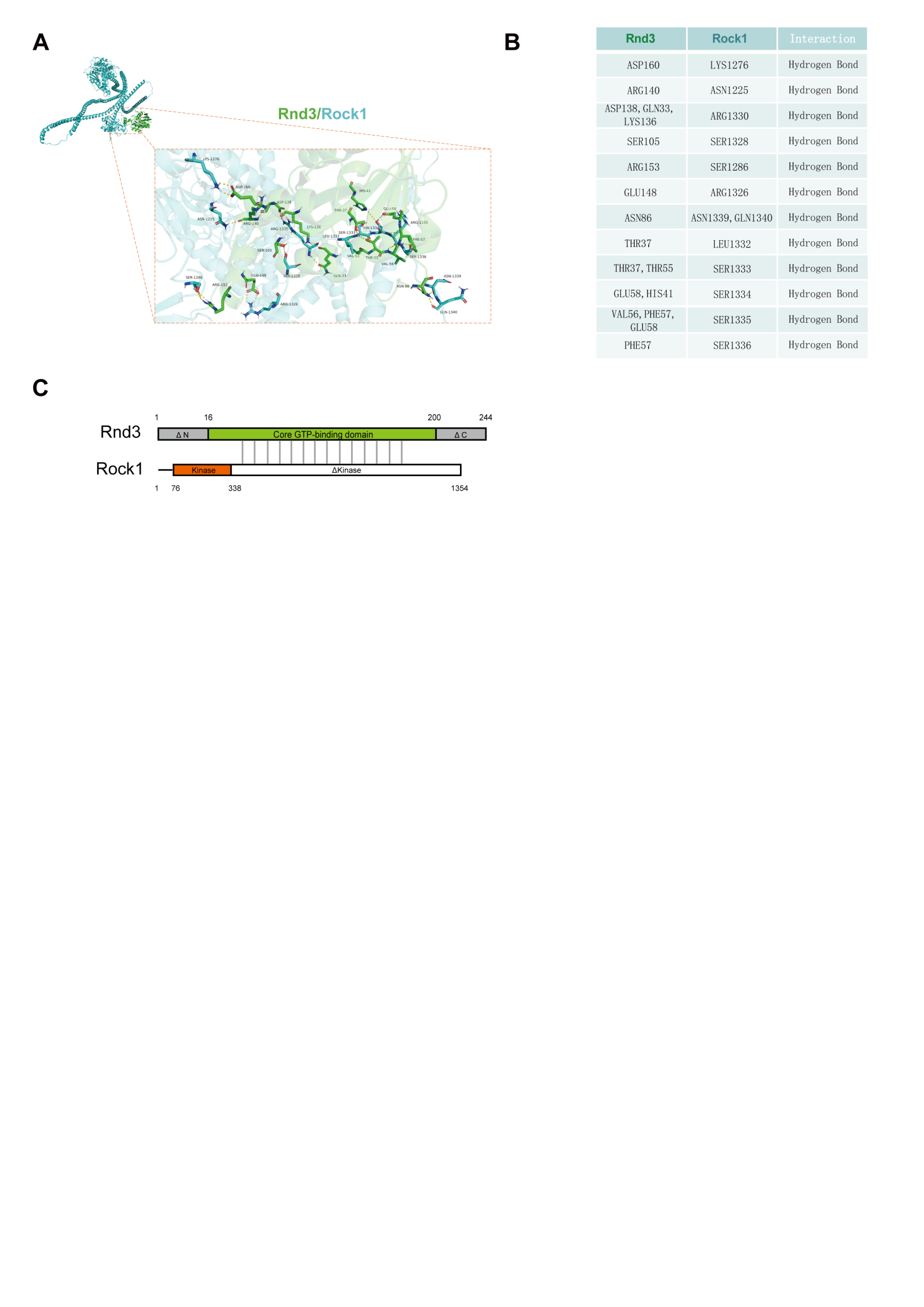
**

**Fig. S10. The combination between Rnd3 and Rock1.**

**A** Structure-based protein interaction interface analysis between Rnd3 and Rock1 using the Cluspro server. **B** The docking sites of Rnd3 and Rock1. **C** Schematic representation of the combination between Rnd3 and Rock1 mutants domain in this study. Full-length Rnd3 has N-terminal extensions (Rnd3ΔC, residues 1-16), a core GTP-binding domain (Rnd3 Core domain, residues 16-200) and C-terminal extensions (ΔC, residues 200-244). Full-length Rock1 has Rock1 Kinase (residues 1-338) and Rock1ΔKinase (residues 338-1354).

**
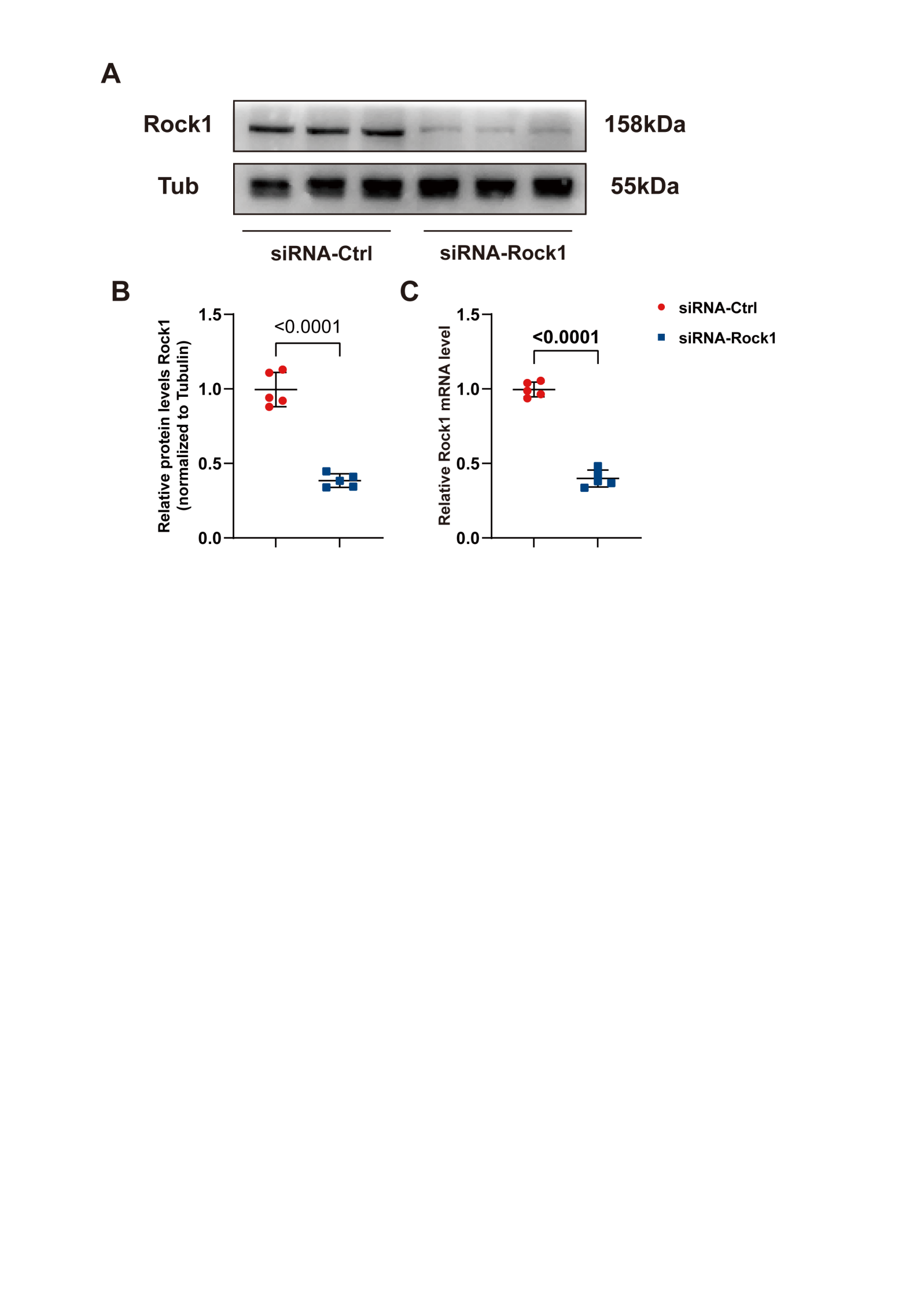
**

**Fig. S11. The efficiency of Rock1 knockdown in cardiomyocytes.**

**A** Representative Western blot images of Rock1 protein levels. **B** Quantitative analysis of Rock1 protein expression (n = 5). **C** Quantitative analysis of Rock1 mRNA expression (n = 5). Data were presented as mean ± SD. Student’s t-test were used for statistical analysis in **B** and **C**.


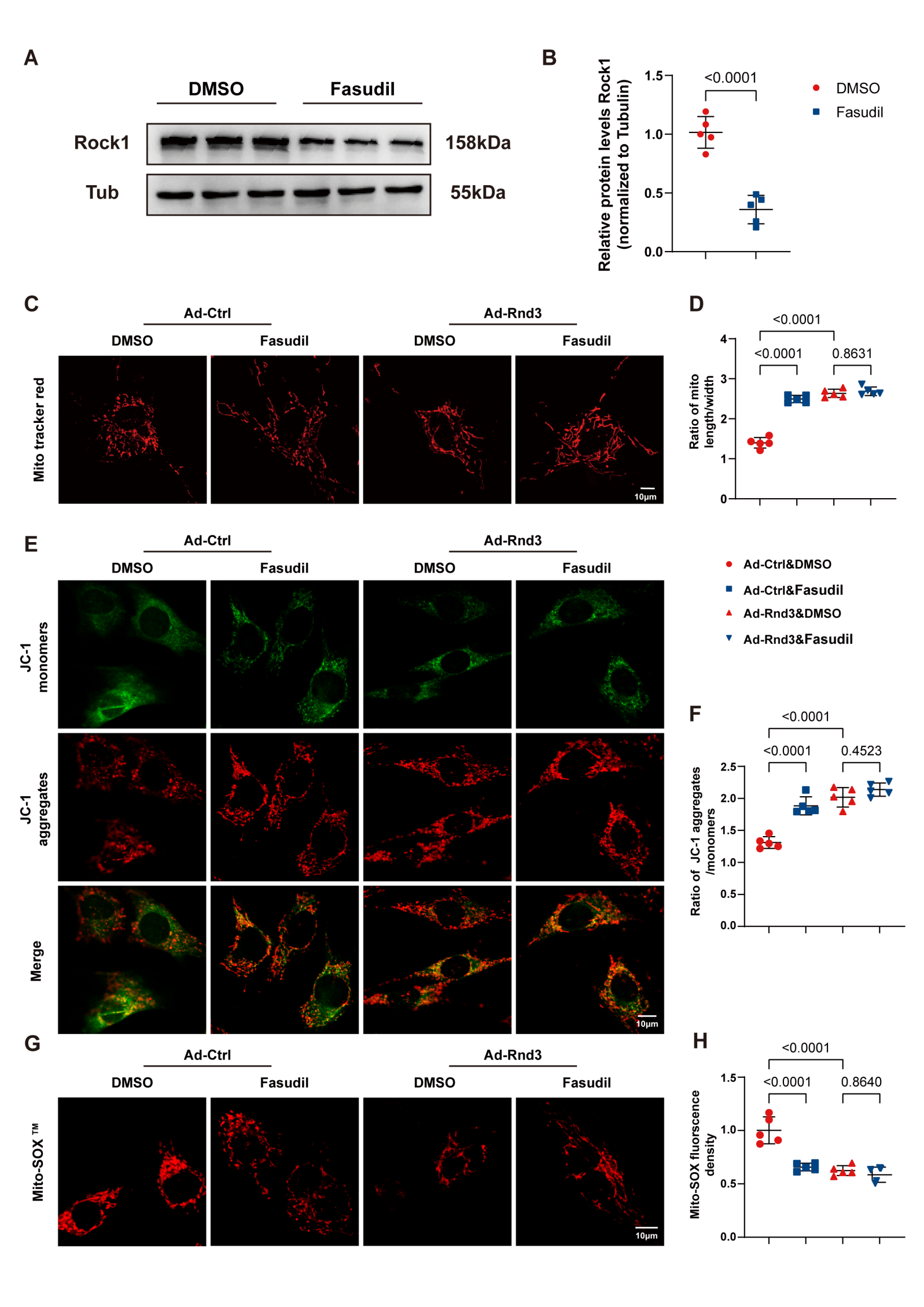


**Fig. S12. Inhibition of Rock1 mediates the Rnd3 protective effect on mitochondrial dysfunction.**

**A** Representative Western blot images of Rock1 levels in cardiomyocytes after following Fasudil. **B** Quantitative analysis of Rock1 protein expression (n = 5). **C** Representative images of mitochondrial morphology in cardiomyocytes following Dox treatment (n = 5). **D** Quantitative analysis of mitochondrial morphology, scale bar = 10μm. **E** Mitochondrial membrane potential measured using the JC-1 assay (n = 5). **F** Quantitative analysis of the ratio of aggregated and monomeric JC-1, scale bar = 10μm. **G** Representative images depicting changes in mitochondrial ROS production in cardiomyocytes (n = 5). **H** Quantitative analysis of mtROS production (n = 5). Data were presented as mean ± SD. Student’s t-test were used for statistical analysis in **B**. One-way ANOVA was used for statistical analysis in **D**, **F**, and **H**.

**
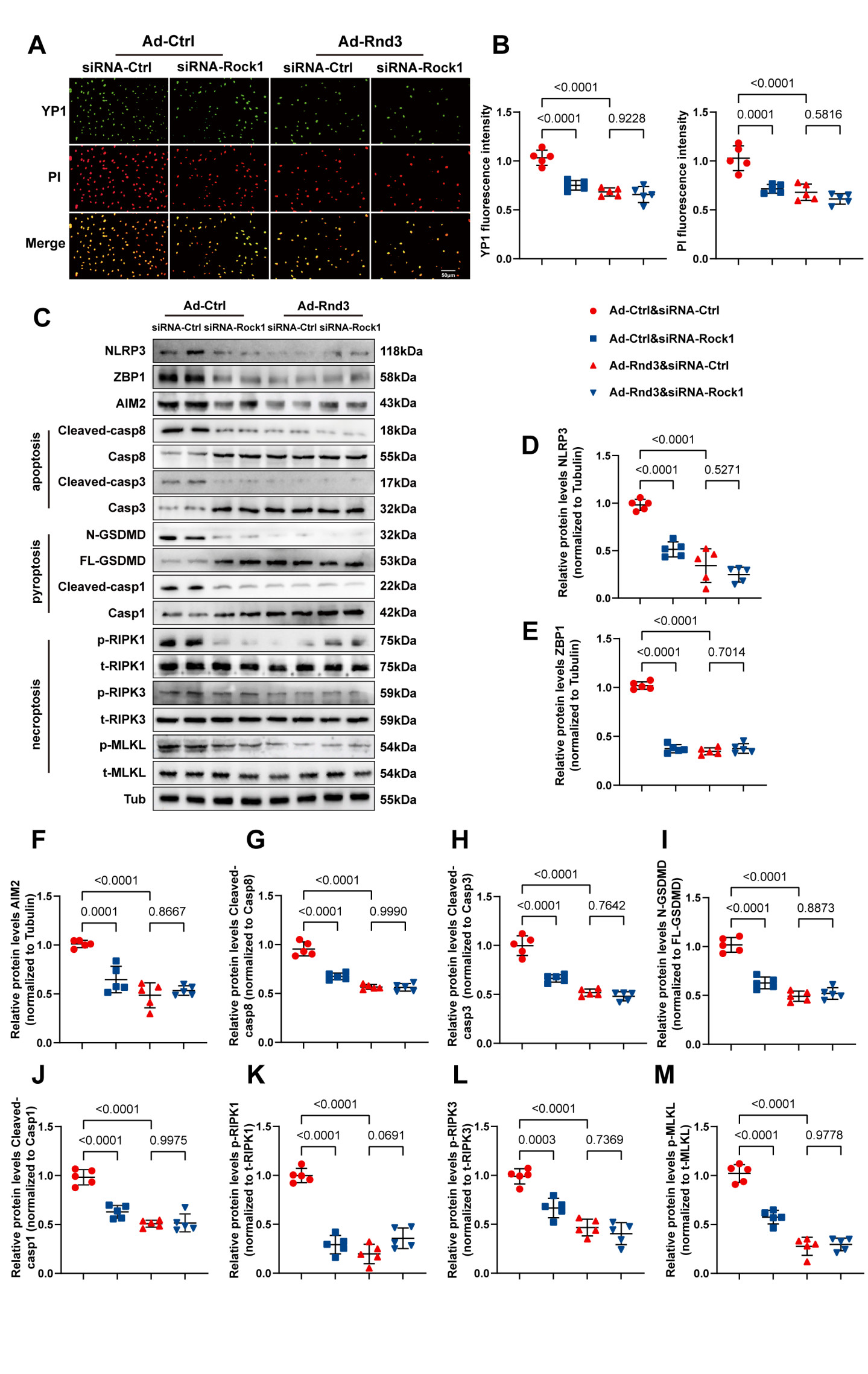
**

**Fig. S13. Rock1 mediates the protective role of Rnd3 against Dox-induced PANoptosis.**

**A** Representative immunofluorescence images showing YP1-positive cells (green) and PI-positive cells (red). Cardiomyocytes were infected with Ad-Control or Ad-Rnd3, and then infected with siRNA-Control or siRNA-Rock1 for 24 h with a 24h exposure to 2µM Dox ensued, scale bar = 50 μm. **B** Quantitative analysis of YP1-positive cells and PI-positive cells (n = 5). **C** Representative Western blot images of PANoptosis-related proteins levels. **D-M** Quantitative analysis of PANoptosis-related proteins expression (n = 5). Data were presented as mean ± SD. One-way ANOVA was used for statistical analysis in **B**, **D**, **E**, **F**, **G**, **H**, **I**, **J**, **K**, **L**, and **M**.

**
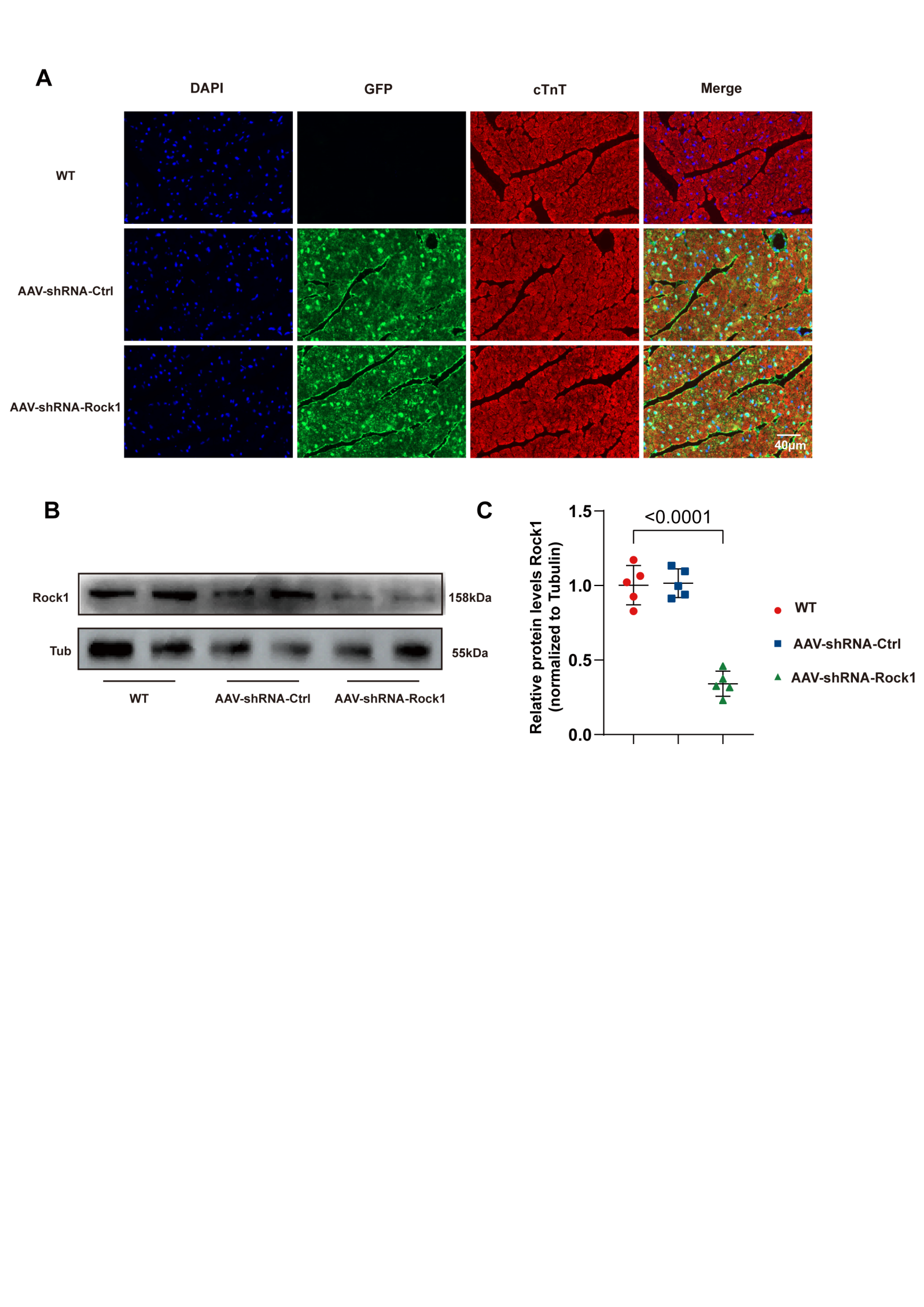
**

**Fig. S14. The efficiency of Rock1 knockdown after intramyocardial injection of AAV-shRNA-Ctrl or AAV-shRNA-Rock1.**

**A** Representative immunofluorescence images of Rock1 in cardiac tissues. **B** Representative Western blot images of Rock1 protein levels in cardiac tissues. **C** Quantitative analysis of Rock1 protein expression (n = 5). Data were presented as mean ± SD. One-way ANOVA was used for statistical analysis in **C**.


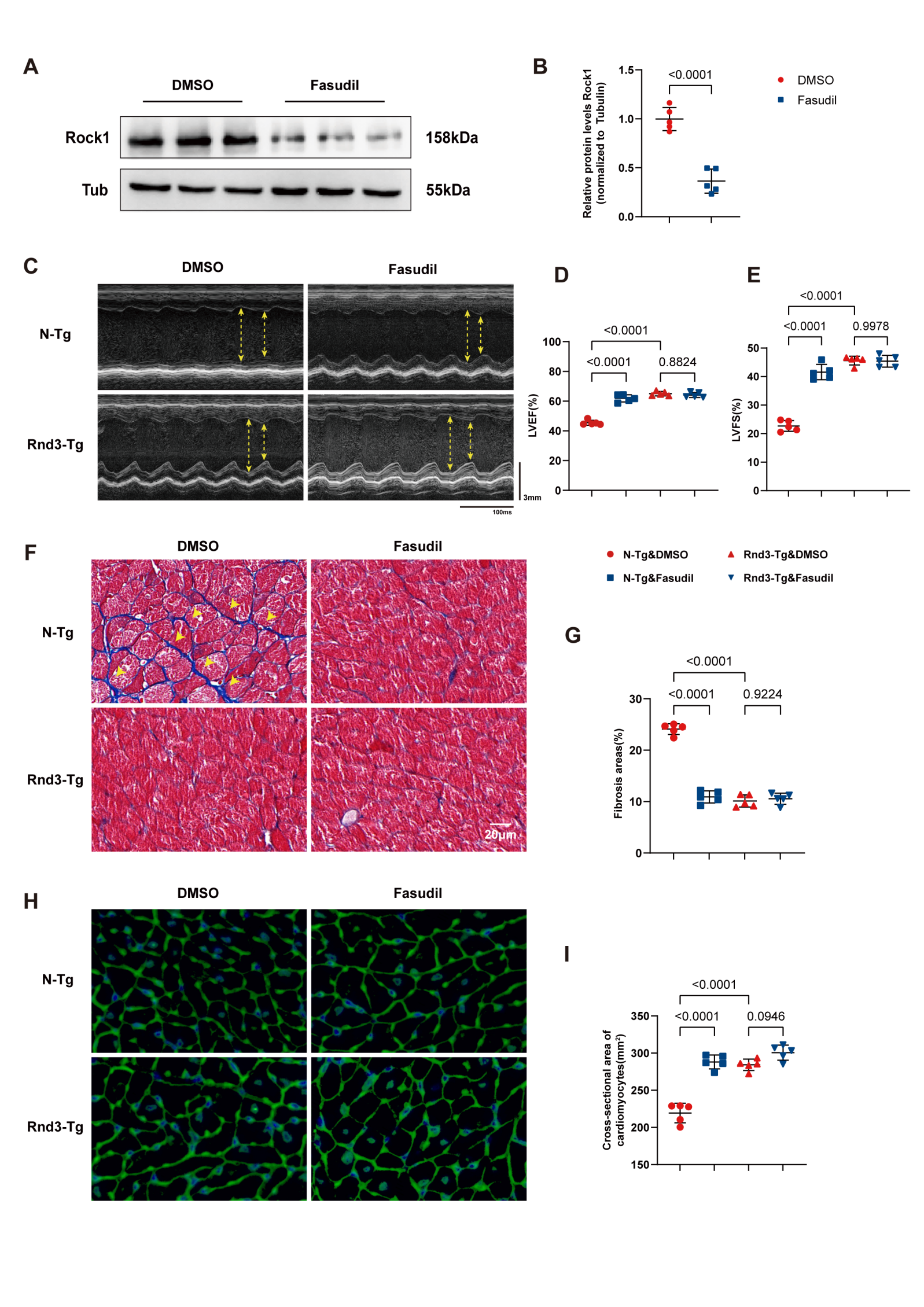


**Fig. S15. The pharmacologic target of Rock1, Fasudil, largely abrogates Rnd3-induced cardioprotective benefits in DIC.**

**A** Representative Western blot images of Rock1 protein levels. **B** Quantitative analysis of Rock1 protein expression (n = 5)**. C** Representative M-mode echocardiographic imaging of heart in mice. **D-E** Analysis of LVEF and LVFS of heart. **F-G** Representative images and quantitative analysis of masson trichrome staining following Fasudil treatment, scale bar = 20 μm. **H-I** Representative WGA staining images and quantification of indicated mice (n = 5). Data were presented as mean ± SD. Student’s t-test were used for statistical analysis in **B**. One-way ANOVA was used for statistical analysis in **D**, **E**, **G** and **I.**
