# Supplementary material for: Rnd3 protects against doxorubicin-induced cardiotoxicity through inhibition of PANoptosis in a Rock1/Drp1/mitochondrial fission-dependent manner
Source: Cell Death Dis. 2025 Jan 4;16(1):2. doi: 10.1038/s41419-024-07322-0 (PMC11700182; doi:10.1038/s41419-024-07322-0)

**Fig.1H**


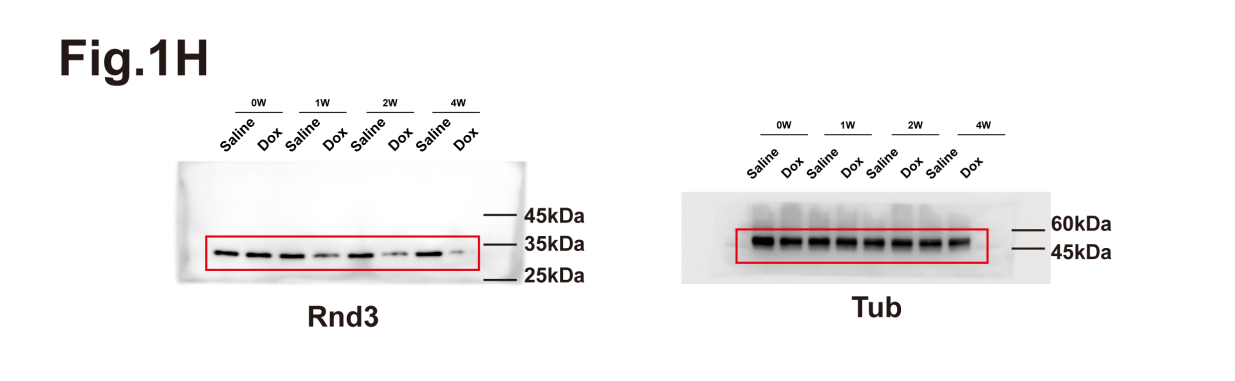


**Fig.3J**


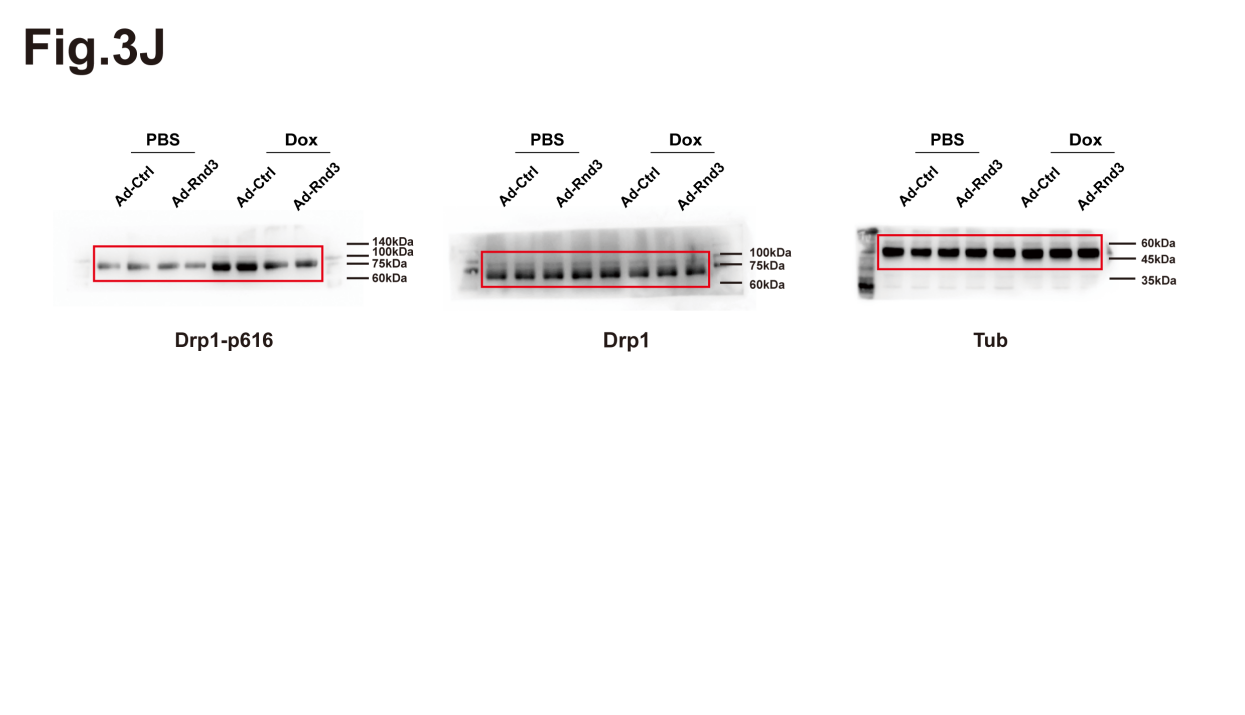


**Fig.4D**
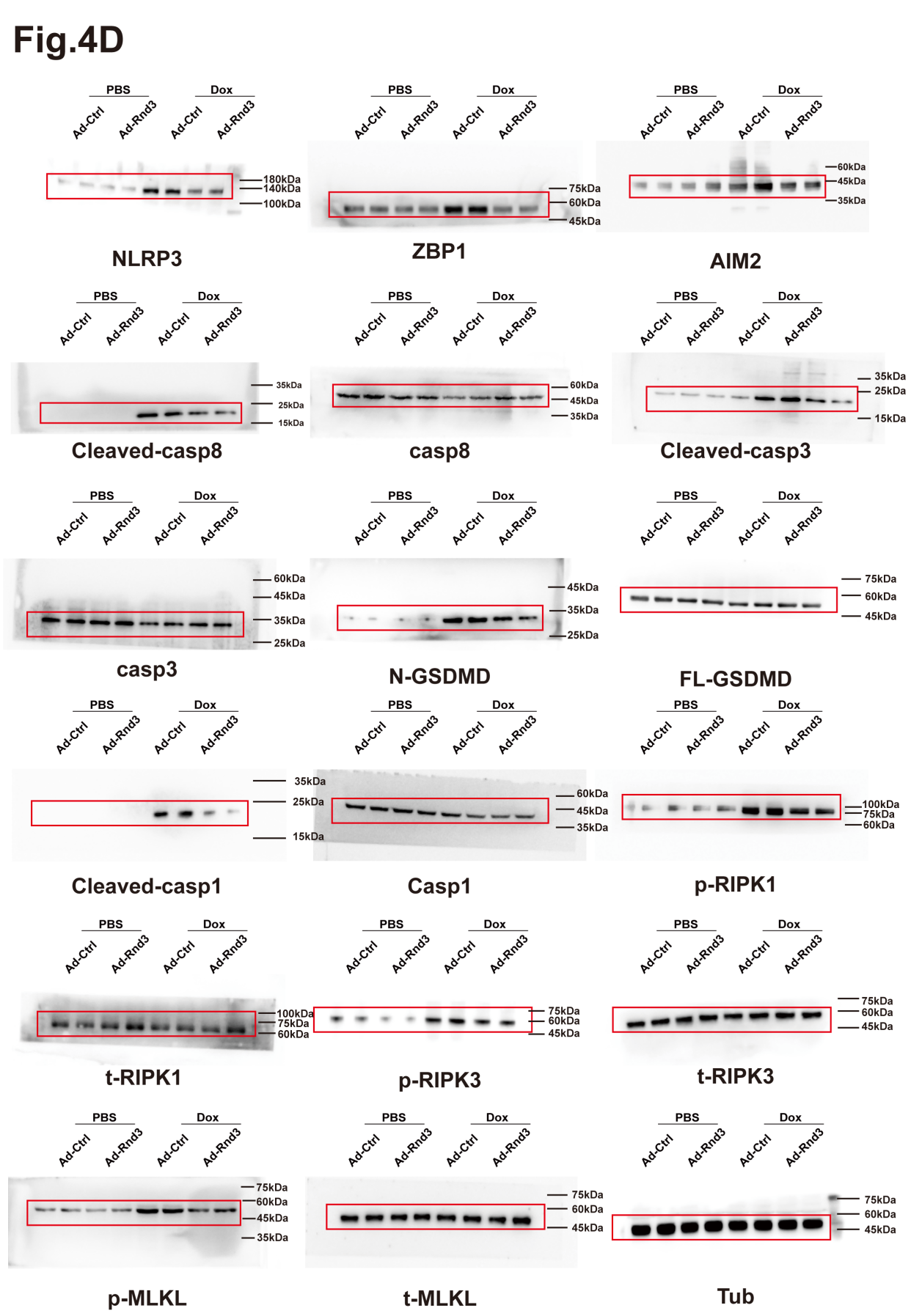


**Fig.5D**

**
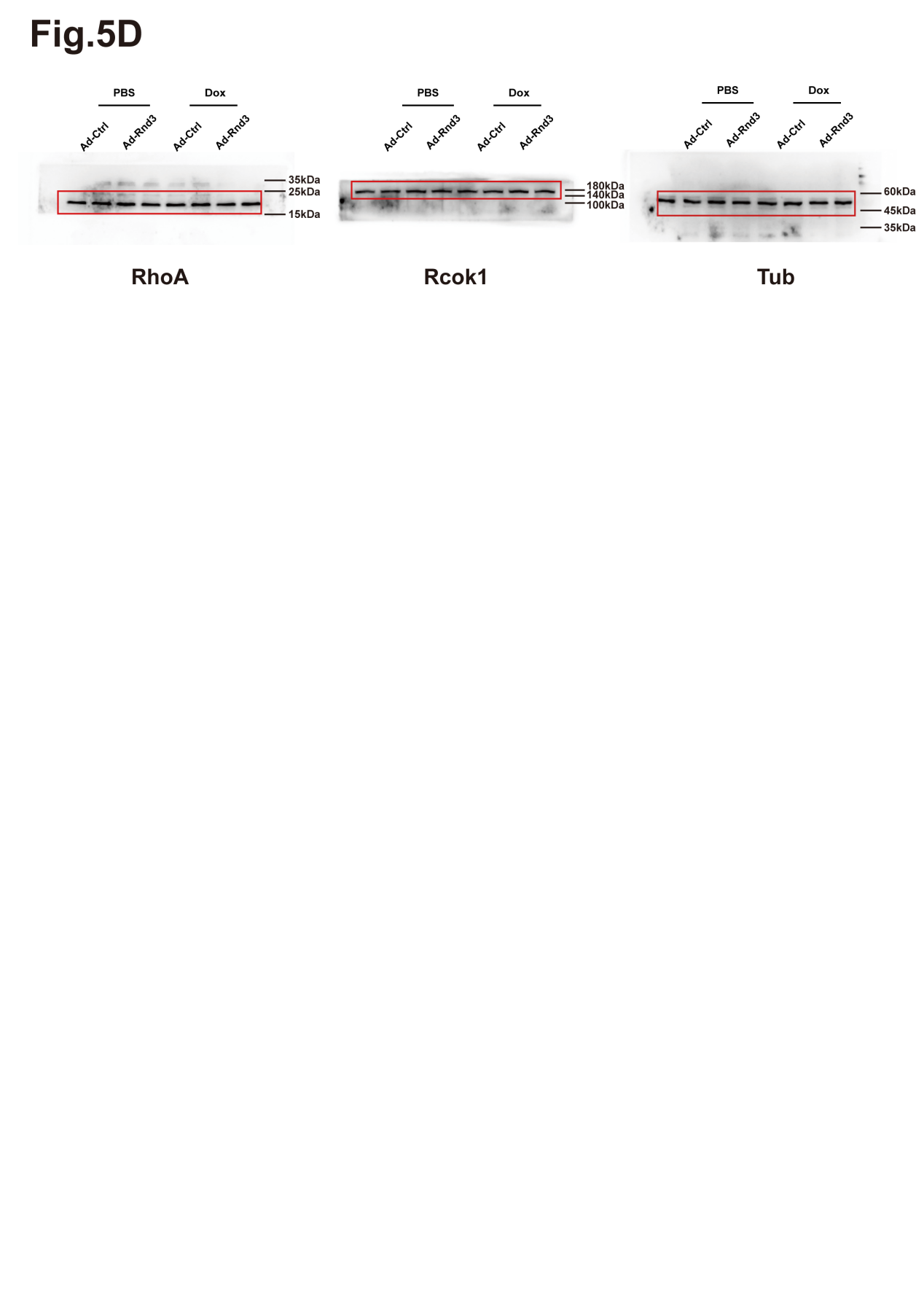
**

**Fig.5G**

**
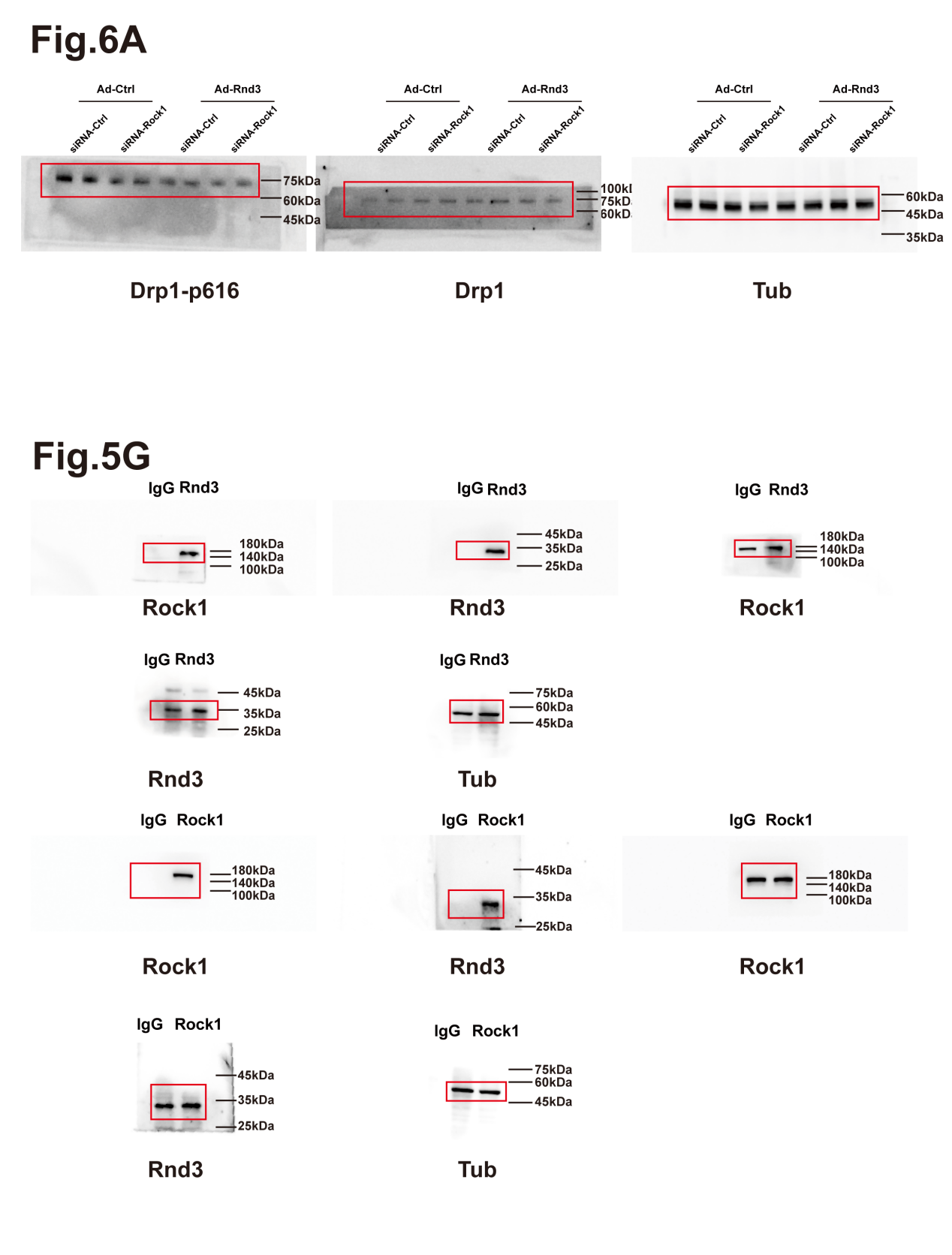
**

**Fig.5J**

**
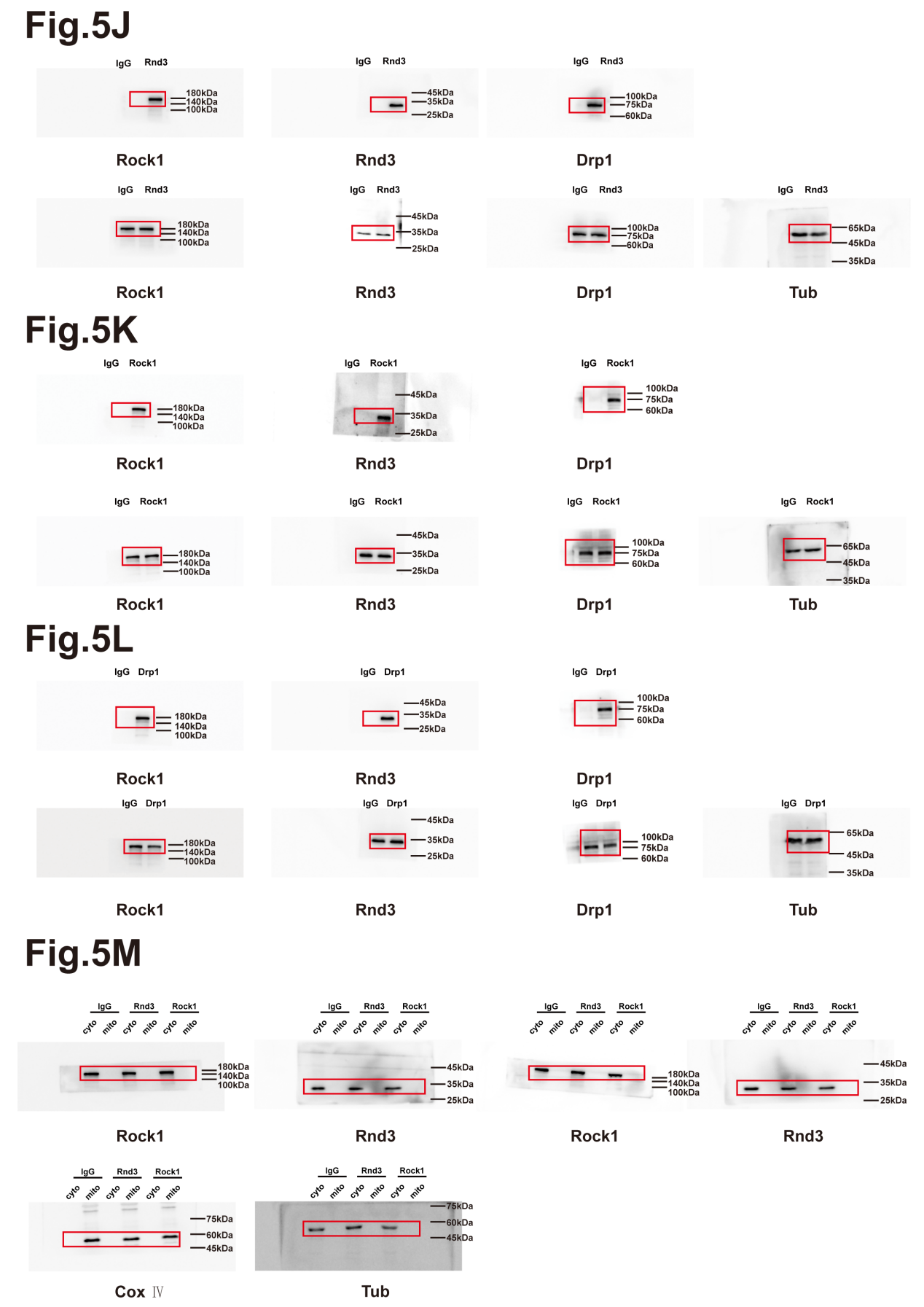
**

**Fig.5K**

**Fig.5L**

**Fig.5M**


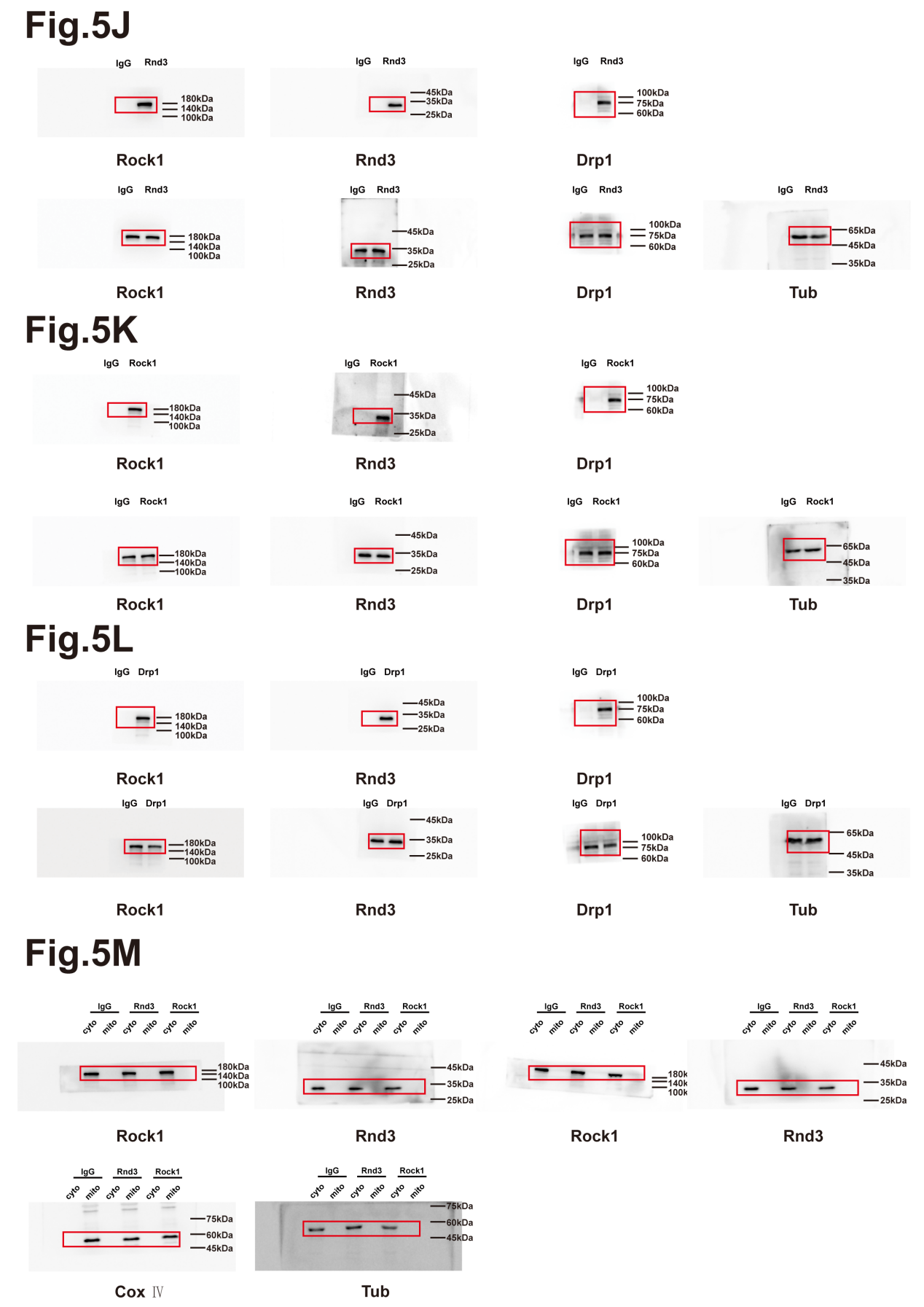


**Fig.6A**


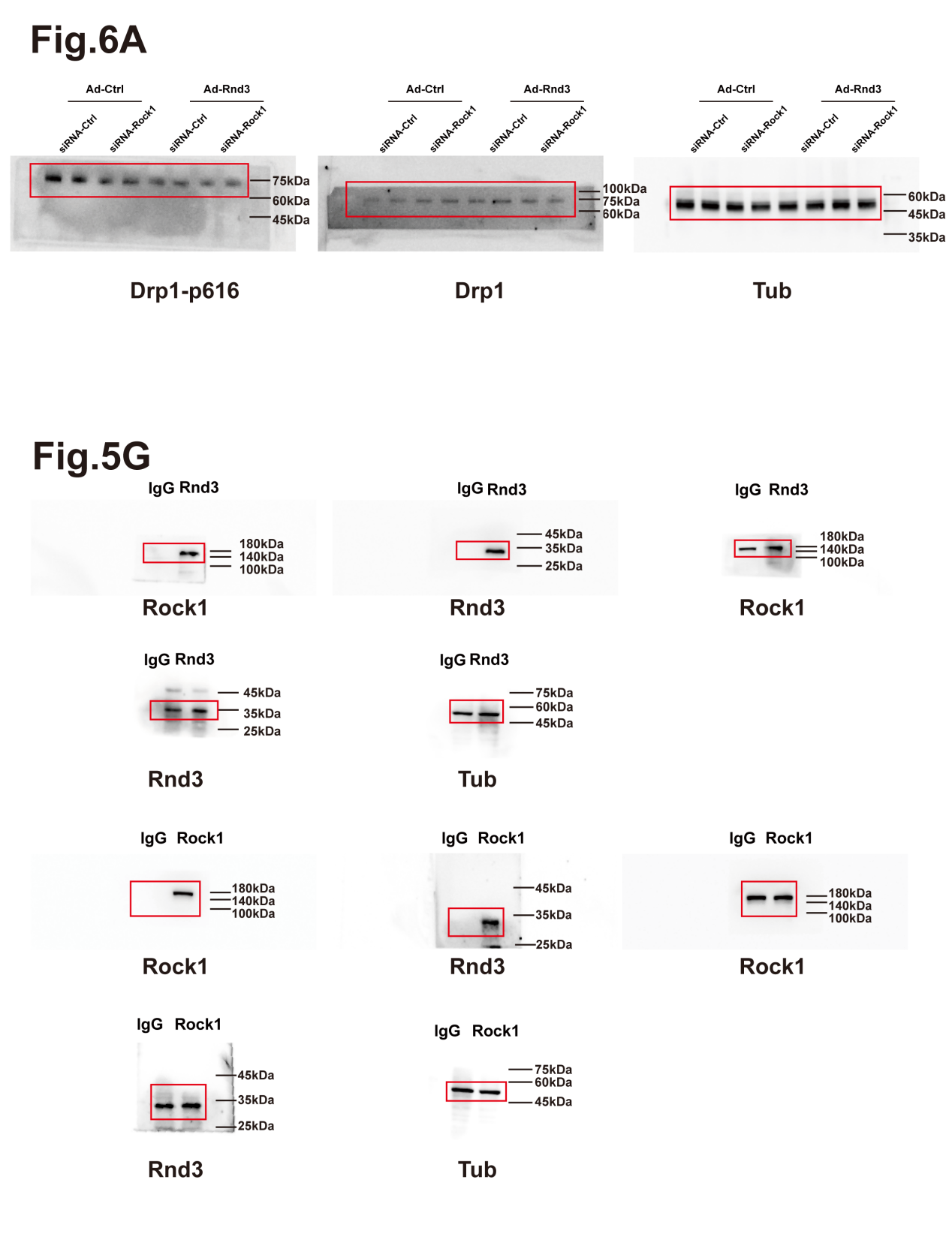


**Fig.S3A**


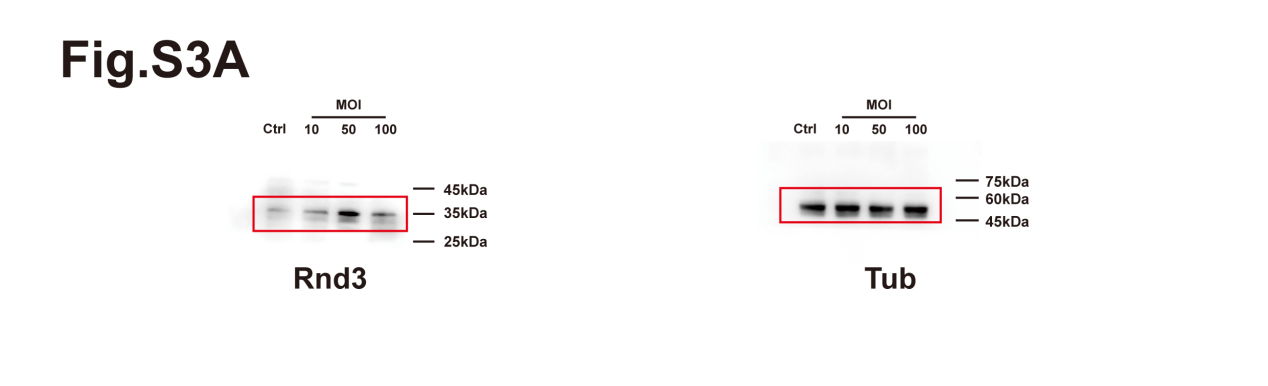


**Fig.S5A**

**
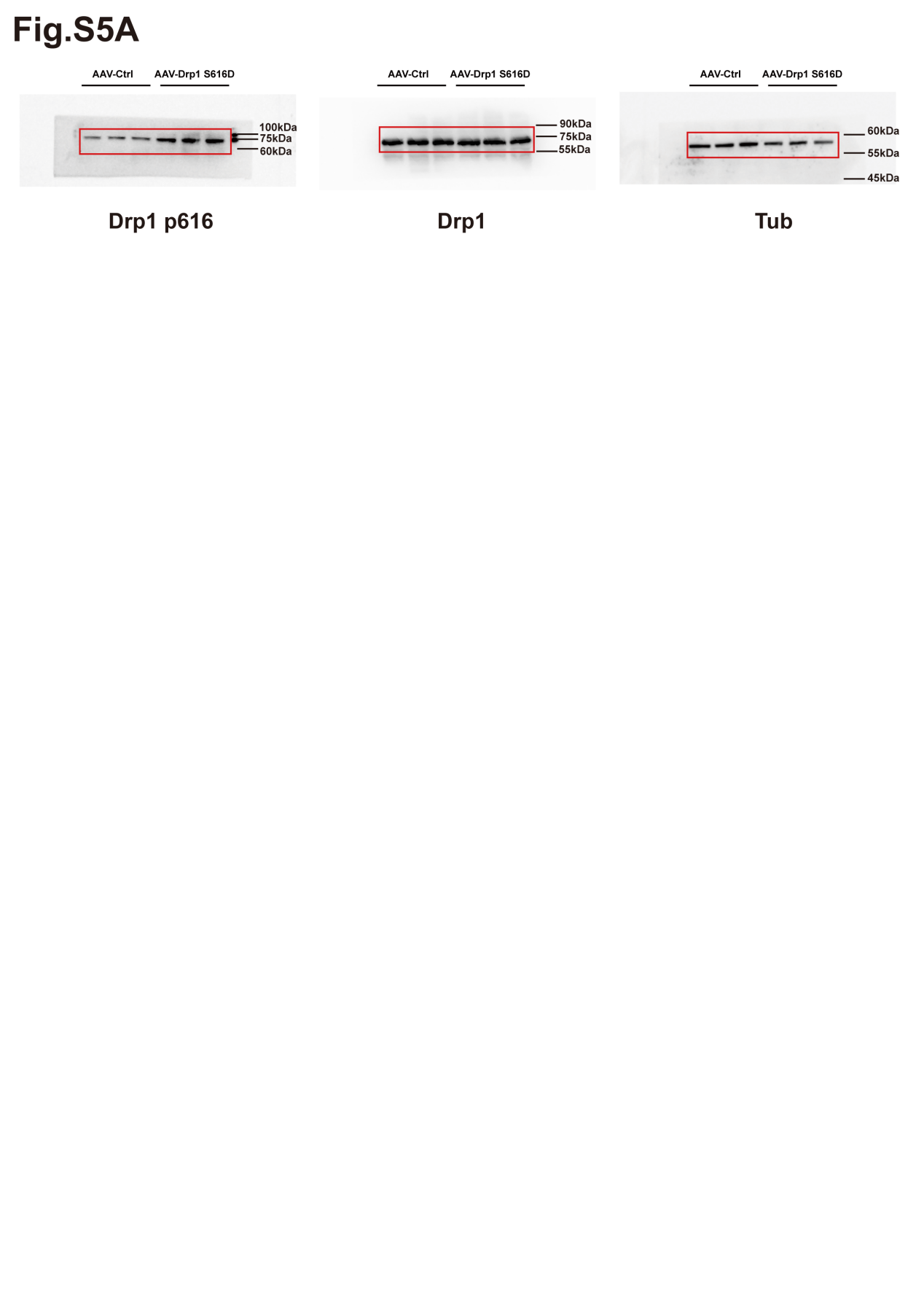
**

**Fig.S7A**

**Fig.S7E**

**Fig.S11A**


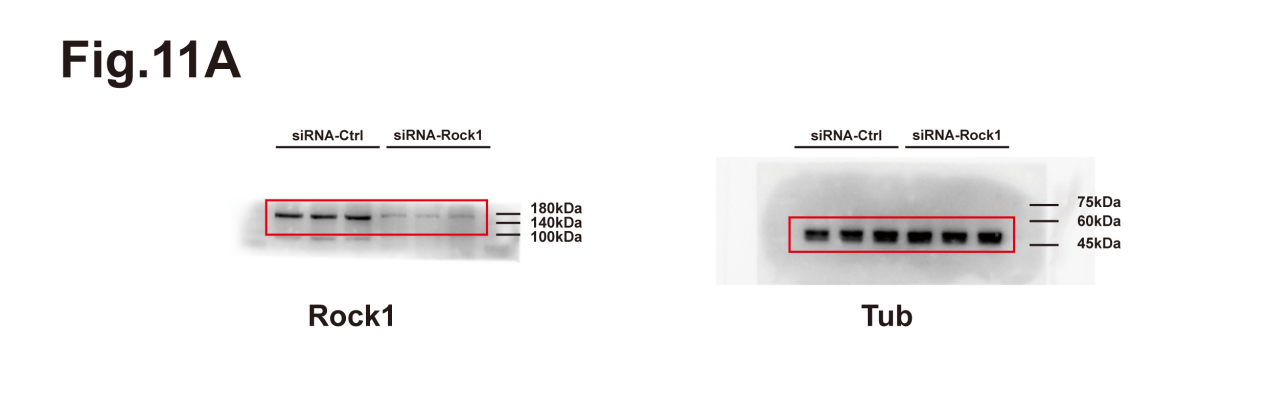


**Fig.S12A**

**
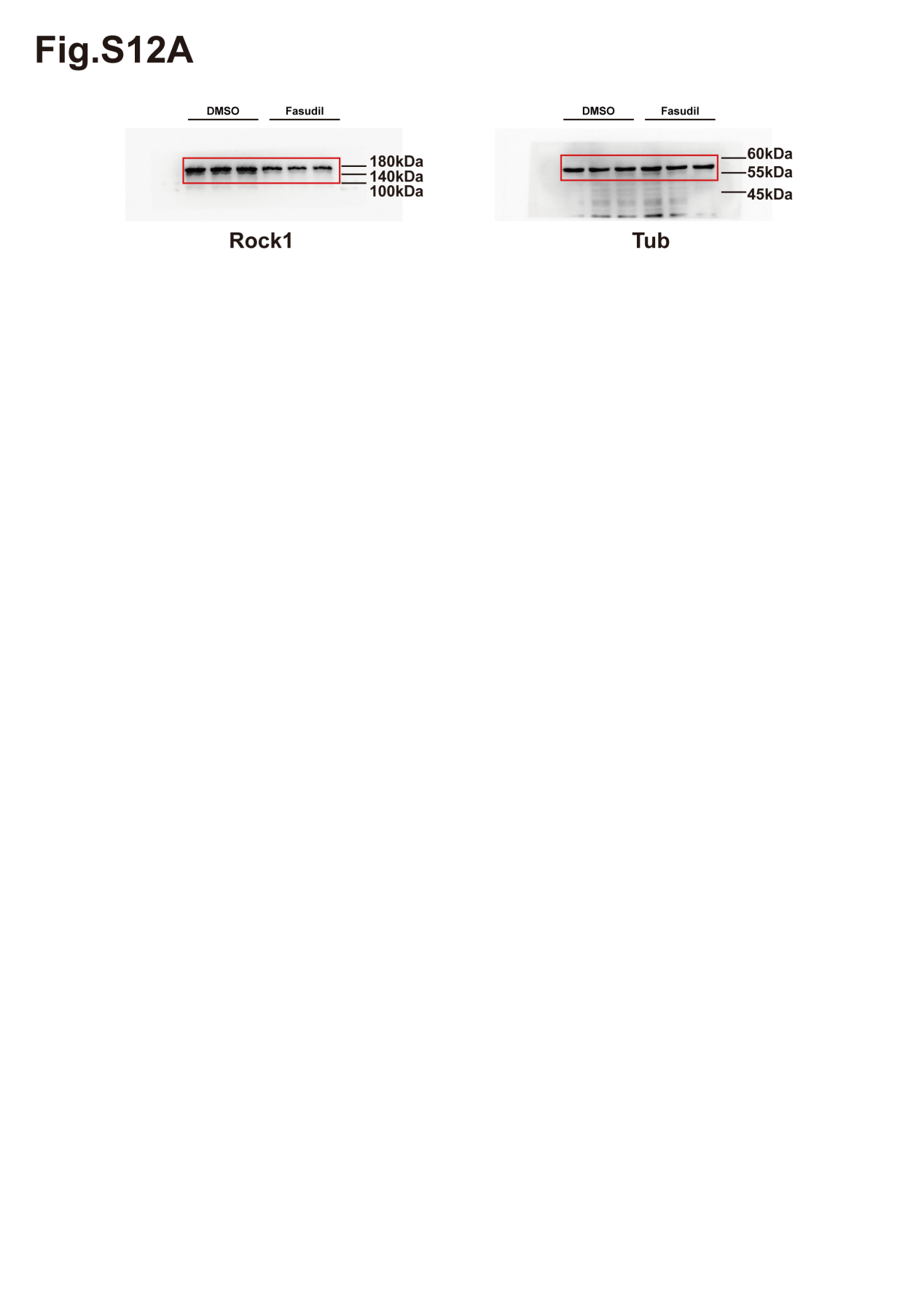
**

**Fig.S13C**


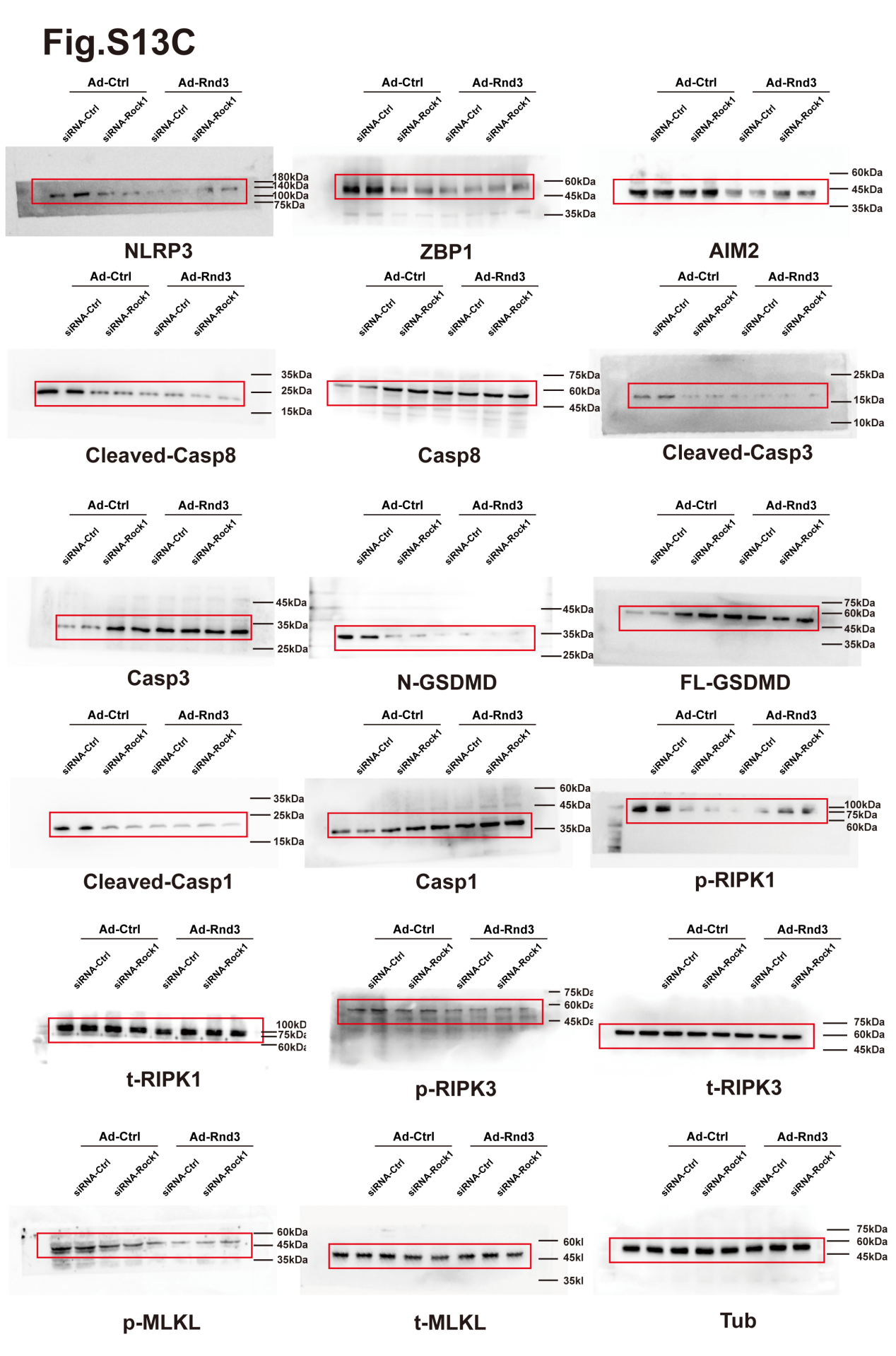


**Fig.S14B**


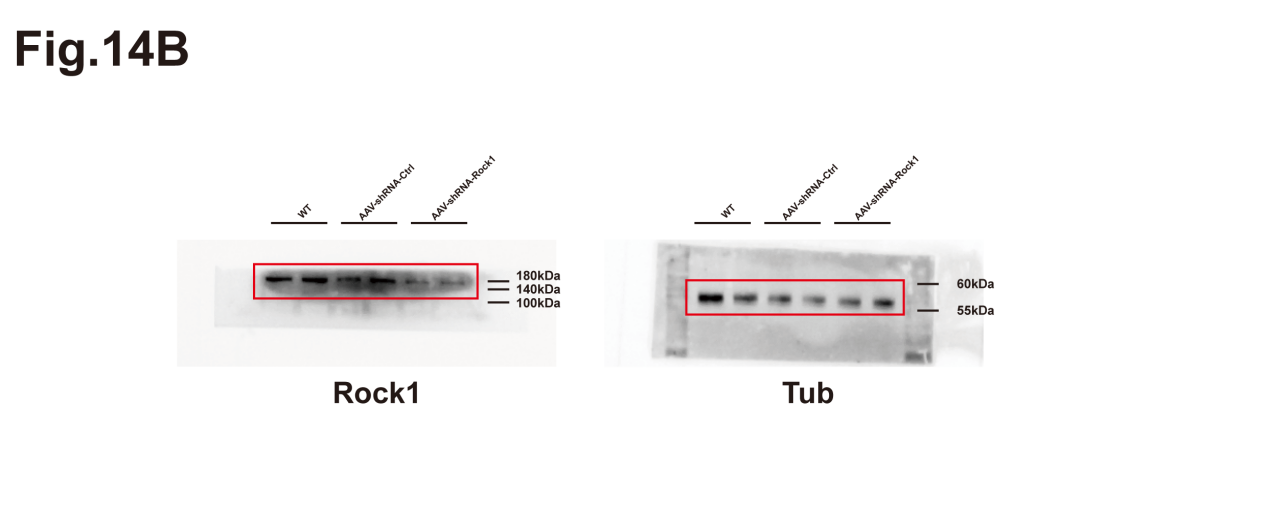


**Fig.S15A**


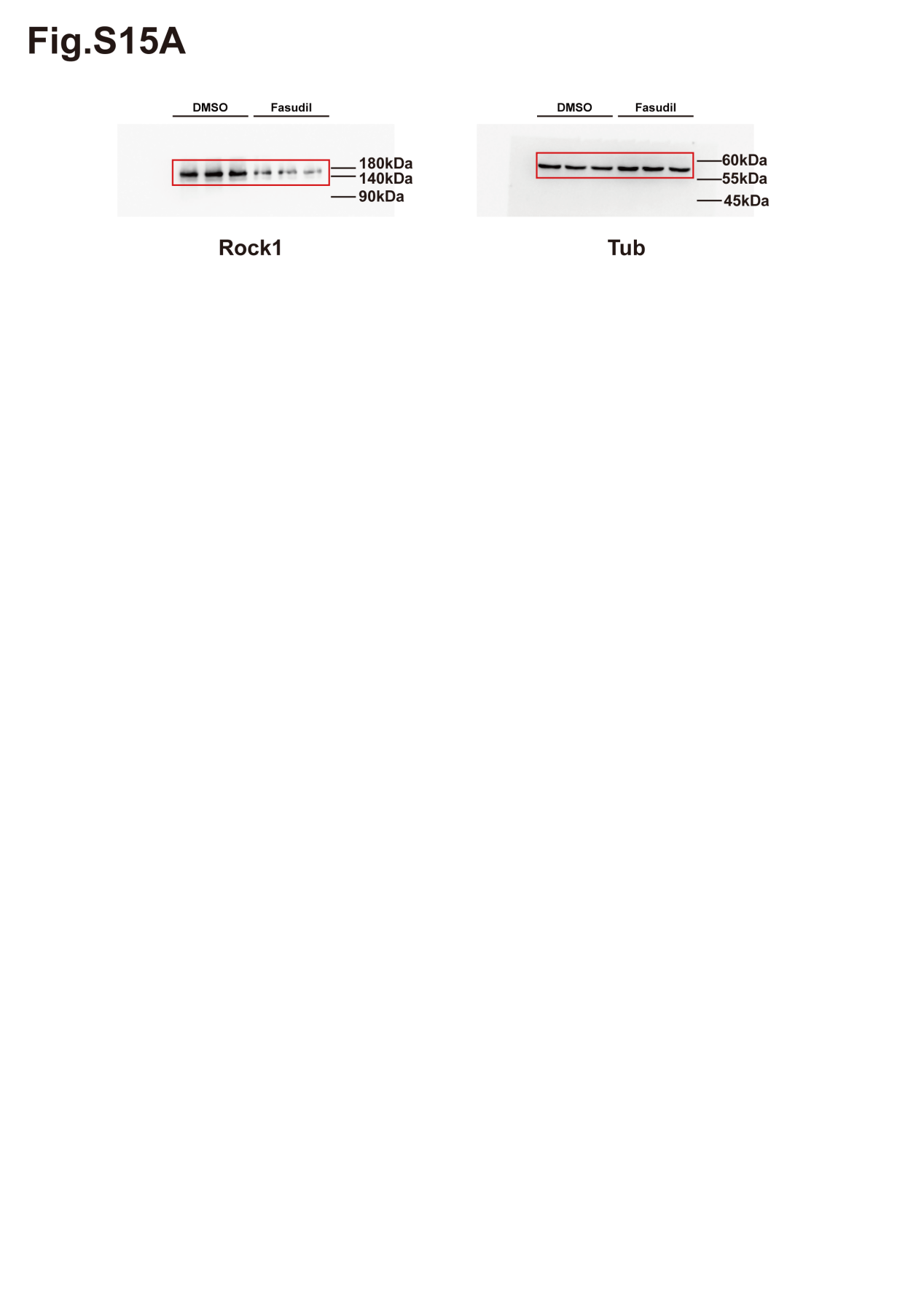

Supplement: Supplementary file 5 — The original Western blot bands [file 41419_2024_7322_MOESM5_ESM.docx]
